# Adaptive aspects of impulsivity and interactions with effects of catecholaminergic agents in the 5-choice serial reaction time task: implications for ADHD

**DOI:** 10.1007/s00213-021-05883-y

**Published:** 2021-06-09

**Authors:** Chiara Toschi, Mona El-Sayed Hervig, Parisa Moazen, Maximilian G. Parker, Jeffrey W. Dalley, Ulrik Gether, Trevor W. Robbins

**Affiliations:** 1 Department of Psychology and Behavioural and Clinical Neuroscience Institute, University of Cambridge, Downing St., CB2 3EB, Cambridge, UK; 2 Department of Neuroscience, University of Copenhagen, DK-2200 Copenhagen, Denmark; 3 Department of Physiology, Faculty of Medical Sciences, Tarbiat Modares University, Tehran, Iran; 4 Department of Psychiatry, University of Cambridge, Cambridge Biomedical Campus, Cambridge, UK

**Keywords:** Impulsivity, Methylphenidate, Atomoxetine, Atipamezole, Amphetamine, Phenylephrine

## Abstract

**Background:**

Work in humans has shown that impulsivity can be advantageous in certain settings. However, evidence for so-called functional impulsivity is lacking in experimental animals.

**Aims:**

This study investigated the contexts in which high impulsive (HI) rats show an advantage in performance compared with mid- (MI) and low impulsive (LI) rats. We also assessed the effects of dopaminergic and noradrenergic agents to investigate underlying neurotransmitter mechanisms.

**Methods:**

We tested rats on a variable inter-trial interval (ITI) version of the 5-choice serial reaction time task (5CSRTT). Rats received systemic injections of methylphenidate (MPH, 1 mg/kg and 3 mg/kg), atomoxetine (ATO, 0.3 mg/kg and 1 mg/kg), amphetamine (AMPH, 0.2 mg/kg), the alpha-2a adrenoceptor antagonist atipamezole (ATI, 0.3 mg/kg) and the alpha-1 adrenoceptor agonist phenylephrine (PHEN, 1 mg/kg) prior to behavioural testing.

**Results:**

Unlike LI rats, HI rats exhibited superior performance, earning more reinforcers, on short ITI trials, when the task required rapid responding. MPH, AMPH and ATI improved performance on short ITI trials and increased impulsivity in long ITI trials, recapitulating the behavioural profile of HI. In contrast, ATO and PHEN impaired performance on short ITI trials and decreased impulsivity, thus mimicking the behavioural profile of LI rats. The effects of ATO were greater on MI rats and LI rats.

**Conclusions:**

These findings indicate that impulsivity can be advantageous when rapid focusing and actions are required, an effect that may depend on increased dopamine neurotransmission. Conversely, activation of the noradrenergic system, with ATO and PHEN, led to a general inhibition of responding.

**Supplementary Information:**

The online version contains supplementary material available at 10.1007/s00213-021-05883-y.

## Introduction

Impulsivity is a multifactorial construct more generally understood as the tendency to act prematurely without foresight (Dalley et al. 2011; Evenden 1999; Whiteside and Lynam 2001; Winstanley et al. 2006). It is often regarded as a maladaptive trait and indeed is widely associated with various psychiatric disorders, including drug addiction (de Wit 2009; Jentsch and Taylor 1999; Kollins et al. 2005) and attention-deficit hyperactivity disorder (ADHD, Solanto 2002). However, impulsivity need not be an exclusively dysfunctional trait and may even be advantageous in certain contexts (Dickman 1985; Dickman 1990; Smillie and Jackson 2006; Cools et al. 2005).

Dickman (1990, 2000) advanced the concept of functional impulsivity, that is ‘the tendency to engage in rapid, error-prone information processing when such a strategy is (..) optimal’, (p.101, Dickman 1990). Based on research in human subjects, Dickman concluded that when the experimental task is very simple, the rapid responding typical of ‘high impulsives’ does not lead to a higher rate of errors (Dickman 1985). Similarly, when there is little time available to make a decision, high impulsive individuals respond with greater accuracy than low impulsive individuals (Dickman and Meyer 1988). In line with this early evidence, it was recently shown that trait impulsivity boosts performance in highly rewarding settings (Cools et al. 2005). Similar conclusions on the advantages of impulsivity can be drawn from other contexts, including entrepreneurship (Lerner et al. 2019; Verheul et al. 2015), and creative literature (Lawrence et al. 2008; White and Shah 2011), and are consistent with the recognised role of context in the expression of ADHD (Barkley 2002; Williams and Dayan 2005). Thus, environments encompassing novel, interesting, and fast-paced activities improve ADHD symptoms among young adults (Lasky et al. 2016).

However, despite growing evidence in humans that impulsivity can confer some adaptive advantages, research in experimental animals is lacking. To investigate whether higher levels of impulsivity can be advantageous in certain contexts, we tested rats on the 5-choice serial reaction time task (5CSRTT) and presented them with pseudo-randomly interleaved inter-trial intervals (ITI) of varying durations from relatively short intervals, of 3 and 5 s in duration, to longer intervals, of 7 and 9 s in duration. We predicted that high impulsive (HI) rats, who typically respond prematurely before the occurrence of any light cue, would perform better when the task requires them to respond quickly whilst low impulsive (LI) rats would be impaired. On the contrary, we expected LI rats to have superior performance when the task required animals to wait longer for the stimulus to appear. To further test how performance on rapid trials was affected by context (i.e. the presentation of interleaved slow and rapid trials) and the extent to which HI and LI rats adapt to high-event rate trials, we also evaluated the effects of short trial presentations, with pseudo-randomly interleaved 3 s and 2 s ITIs. The variable ITI (vITI) paradigm offers a range of ITIs and can thus allow different behavioural tendencies to emerge. In addition, the unpredictability in the presentation of each ITI increases attentional load, whilst controlling for the habituation or timing strategies that the animals may adopt as the session progresses (Bizarro et al. 2004; Cope et al. 2016). Whilst other studies have examined performance under vITI versions of the 5CSRTT (Bizarro et al. 2004; Callahan et al. 2019; Carli et al. 1983; Navarra et al. 2008; Paterson et al. 2011; Robinson 2012; Sirviö et al. 1993), none have yet investigated whether HI and LI rats perform differentially during this experimental manipulation and whether a specific impulsivity phenotype confers a selective advantage in performance. Blondeau and Dellu-Hagedorn (2007) tested whether rats segregated on the basis of impulsivity as well as attentional accuracy show a selective advantage in long (8 s) ITI as opposed to short (2 s) ITI trials. Those authors, however, presented trials in isolation as separate challenges and only measured percentage premature responses and percentage correct responses as indexes of performance efficiency. Percentage correct responses, however, does not adequately test whether a specific phenotype has an advantage in performance since it obscures information on incorrect responses and could thus just indicate a more or less prominent response bias in a specific ITI, but not superior performance. In our study, we instead assessed reinforcers earned and omission responses, as well as premature responses, to test the hypothesis that HI and LI rats exhibit different advantages in performance depending on the ITI. Finally, Blondeau and Dellu-Hagedorn (2007) did not test the stability of performance of different impulsivity phenotypes, whilst we tested this on multiple sessions and in two separate batches of animals, thus strengthening the validity of our results.

Additionally, we administered pharmacological agents that are widely used to treat ADHD — d-amphetamine (AMPH), methylphenidate (MPH) and atomoxetine (ATO) — to investigate how different medications affect the performance of animals segregated on the basis of their impulsivity phenotype. Importantly, we chose drugs with different though overlapping effects on catecholamine transmission to more precisely dissect the contribution of distinct neurotransmitter systems in the vITI-5CSRTT paradigm. On the basis of evidence showing that AMPH and MPH impair ‘waiting’ impulsivity (Navarra et al. 2008; Pattij et al. 2007) and decrease response latency (Bizarro et al. 2004), we predicted that administration of these drugs would lead to an improvement of performance in short ITI trials especially. On the contrary, since ATO reduces impulsivity (Blondeau and Dellu-Hagedorn 2007) and slows responding in some contexts (Callahan et al. 2019), we predicted that ATO would mostly enhance performance on long ITI trials. To better dissect the role that noradrenaline (NA) plays in modulating performance of HI and LI rats, we also assessed the effects of systemic administration of atipamezole (ATI), an alpha-2a antagonist, and phenylephrine (PHEN), an alpha1 agonist. To the best of our knowledge, ATI and PHEN have not been tested on animals segregated based on impulsivity; thus, it is unknown how these drugs would interact with this phenotype. In addition, PHEN has never been tested on a vITI paradigm such as the one used in this experiment. On the basis of evidence showing that ATI increases behavioural activation (Ma et al. 2005; Sirviö et al. 1994), whilst PHEN has the opposite effect (Pattij et al. 2012), we predicted that the former would improve performance during short ITI trials, whilst the latter would impair performance.

## Methods and materials

### Subjects

Sixty outbred male Lister Hooded rats (Charles River, Margate, UK) weighing 280–300 g at the beginning of the experiments were used for this study. Animals were acclimatised to the animal facility under a 12 h:12 h light cycle (lights off at 7 AM) for a minimum of 7 days before any procedure began. When rats reached a body weight of approximately 300 g, they were food-restricted to maintain approximately 90% of their free-feeding weight trajectory (19 g of Purina rodent chow per animal and day; adjusted for reward pellet consumption during testing). Water was available ad libitum, and food was given at the end of each day’s testing. All procedures conformed to the UK (1986) Animal (Scientific Procedures) Act (Project licence PA9FBFA9F: Neurobehavioural mechanisms of mental health, held by Dr. A. L. Milton) and were approved by the local Ethics Committee at Cambridge University.

### Behavioural apparatus

Twelve five-hole operant chambers (Med Associates, Georgia, VT) controlled by two computers and Whisker Control software (Cardinal and Aitken 2010) were used. Each chamber was enclosed in a ventilated sound-attenuating box, fitted with five apertures in a curved wall and a food magazine on the opposite wall of the box that delivered rodent sugar pellets (TestDiet®, Purina, UK). A yellow light-emitting diode stimulus was placed at the rear of each aperture. The food magazine and entire chamber were illuminated by light emitting diodes. Infrared beams detected responses in the magazine and apertures.

### Five-choice serial reaction time task: training

All rats were trained in the 5CSRTT as described previously (Bari et al. 2008). Animals were trained to detect a brief visual cue appearing in one of five apertures of the operant chambers. Each trial is initiated when the rat pokes into the food magazine, and the visual cue is presented after an ITI of 5 s. A response was deemed ‘correct’ if the animal poked into the hole where the light was presented within 5 s of target presentation. A nose-poke response occurring before the appearance of the visual cue was considered ‘premature’, whilst a response occurring in any of the apertures where the light was not presented was considered ‘incorrect’. A failure to respond within 5 s of target presentation was recorded as an ‘omission’ of response. Only correct responses were rewarded with a food pellet (Noyes dustless pellets, Research Diets, UK), whilst incorrect, premature and omission responses were punished with a time-out period of 5 s. During a time-out, the animal was required to wait for the beginning of the next trial in order to engage again with the task. Nose-pokes in any of the holes made after a correct or incorrect response, but prior to reward collection, were deemed ‘perseverative’ but were not signalled by punishment. Each session lasted a maximum of 100 trials or 30 min, whichever limit was reached first. During the training session, stimulus duration was set at 30 s and was gradually decreased over sessions until animals reached stable baseline performance (accuracy, >80% correct choice and <20% errors of omission). In Experiment 1, thirty-six animals were trained to reach a stable baseline performance on the 5CSRTT with a final stimulus duration of 0.7 s and an ITI of 5 s. In Experiment 2, twenty-four animals were trained to reach a stable baseline performance on the 5CSRTT with a final stimulus duration of 0.6 s and an ITI of 5 s.

### Experiment 1: Effects of impulsivity trait on behavioural performance at variable ITIs

#### Variable ITI challenge

Thirty-six rats reached a stable baseline performance and were subsequently exposed to two vITI sessions. Each vITI session was followed by at least 1 day of baseline testing where rats were run according to the baseline parameters specified above. Each vITI session consisted of a pseudo-random presentation of trials with 3 s, 5 s, 7 s, and 9 s ITI (mean of 6 s). Each ITI was presented at least 50 times, and the session ended when animals had completed 200 trials or after 2 h (whichever event occurred first). Animals could not predict which ITI was going to be presented on each trial. Time-out (0.5 s) and stimulus duration (0.7 s) were kept constant at the same level as their baseline training. To identify which animals exhibited extreme impulsivity phenotypes, rats underwent an impulsivity screening procedure. Specifically, premature responses across the 2 days of vITI challenge were averaged, and the upper (i.e. the 9 highest-impulsive rats) and lower (i.e. the 9 lowest-impulsive rats) were selected. Animals falling between these two extremes were classified as mid-impulsive (MI) rats.

#### Short vITI challenge: rapid stimulus presentation

A day after their last vITI challenge, rats were presented with a short vITI session. This consisted of 100 trials of 3 s ITI and 50 trials of 2 s ITI (mean of 2.6 s). The two different ITI trials were pseudo-randomly interleaved, and the rat could not predict which ITI trial was going to be presented. The session ended when rats had completed 150 trials or after 1 h and 30 min (whichever occurred first). More instances of the 3 s ITI were presented compared to the 2 s ITI, to avoid making the task too difficult (and risk having floor effects), whilst still exploring whether rats could be challenged with even quicker ITI trials than 3 s and whether trait impulsivity influenced performance on these two ITIs differently.

### Experiment 2: Effects of methylphenidate, atomoxetine, amphetamine, atipamezole and phenylephrine on vITI performance

#### Variable ITI challenge

Twenty-four rats were exposed to three vITI sessions similar to those of Experiment 1. Each vITI session was followed by at least 1 day of baseline testing with the baseline parameters specified above. Each vITI session consisted of a pseudo-random presentation of trials with 3 s, 5 s, 7 s and 9 s ITI. Each ITI was presented at least 50 times; the session ended when animals had completed 200 trials or when 2 h had passed (whichever occurred first). Animals could not predict which ITI was going to be presented on each trial. Time-out (5 s) and stimulus duration (0.6 s) were kept constant at the same level as that of their baseline training. To identify which animals exhibited extreme impulsivity phenotypes, rats underwent a screening procedure. Specifically, premature responses across the 3 days of vITI challenge were averaged, and the upper (*N* = 6) and lower (*N* = 6) quartiles were selected. Animals falling between these two extremes were deemed MI impulsive rats. Following this challenge, rats were also tested on a fixed 7 s ITI session to compare behaviour on the vITI challenge with previous studies on impulsive responding using a fixed 7 s ITI procedure.

#### Systemic drug administration

All rats (HI, MI and LI) received control injections of the vehicle 2 days before the start of the experiment. All drugs were administered sub-cutaneously (s.c.) 40 min prior to testing. The drug experiments consisted of two separate randomised within-subject cross-over Latin-square designs, to control for training and crossover effects. These two Latin-square designs were separated by at least 3 days of washout. In Latin-square 1, vehicle, MPH (1 mg/kg and 3 mg/kg) and ATO (0.3 mg/kg and 1 mg/kg) were administered. In Latin-square 2, vehicle, AMPH (0.2 mg/kg), ATI (0.3 mg/kg) and PHEN (1 mg/kg) were administered. All drugs were dissolved in 0.9% saline and vehicle consisted of just 0.9% saline. Drugs were tested on vITI challenges only (mean ITI of 6 s).

#### Data analysis

The main dependent variables were percentages of premature responses, percentages of omission responses, the number of reinforcers earned and response latencies to make correct, incorrect or premature responses. To assess the temporal profile of responses, we divided the vITI sessions into 5-min bins. Each bin had to have responses from at least three animals from each impulsivity group to be included in the analyses. The 5-min bins satisfying this criteria, across different sessions and cohorts of animals, were from 5 to 55 min (11 bins).

Percentages, number of reinforcers and the number of active responses per unit of time were square root transformed. Latencies were log-transformed. Transformations were applied to enable comparisons with previous publications (Hervig et al. 2020; Milstein et al. 2010) and to avoid incurring issues of non-normal data distributions. Statistical tests were performed with RStudio, version 1.2.1335 (RStudio, Inc). Data were subjected to Linear Mixed-Effects Model analysis with the lmer package in R. To validate whether the data transformations improved model fit, we compared the AIC values of the models with transformed and non-transformed data. The model with transformed data yielded the lowest AIC values for all variables. For analyses of behaviour prior to any drug manipulation, the model contained three fixed factors (day, ITI and impulsivity) and one factor (subject) modelled as a random slope to account for individual differences between rats across testing days. When significant three-way interactions were found, further analysis was performed by conducting separate multilevel models on ‘day’. For analyses of drug interventions, the model contained three fixed factors (ITI, impulsivity and drug) and one factor (subject). When significant three-way interactions were found, further analysis was performed by conducting separate multilevel models on ‘impulsivity’. For all analyses, significance was considered at *α* = 0.05. When significant interactions were found, further analysis was performed by conducting post hoc Tukey’s corrected pairwise comparisons. For drug manipulations, post-hoc testing was used to determine differences with vehicle treatment only.

## Results

Baseline performance prior to the vITI challenge was analysed and is reported in detail in the supplementary materials. Briefly, on baseline, HI rats exhibited elevated premature responses compared with the other two groups.

### Experiment 1: Effects of impulsivity trait on behavioural performance at variable ITIs

**Fig. 1:**
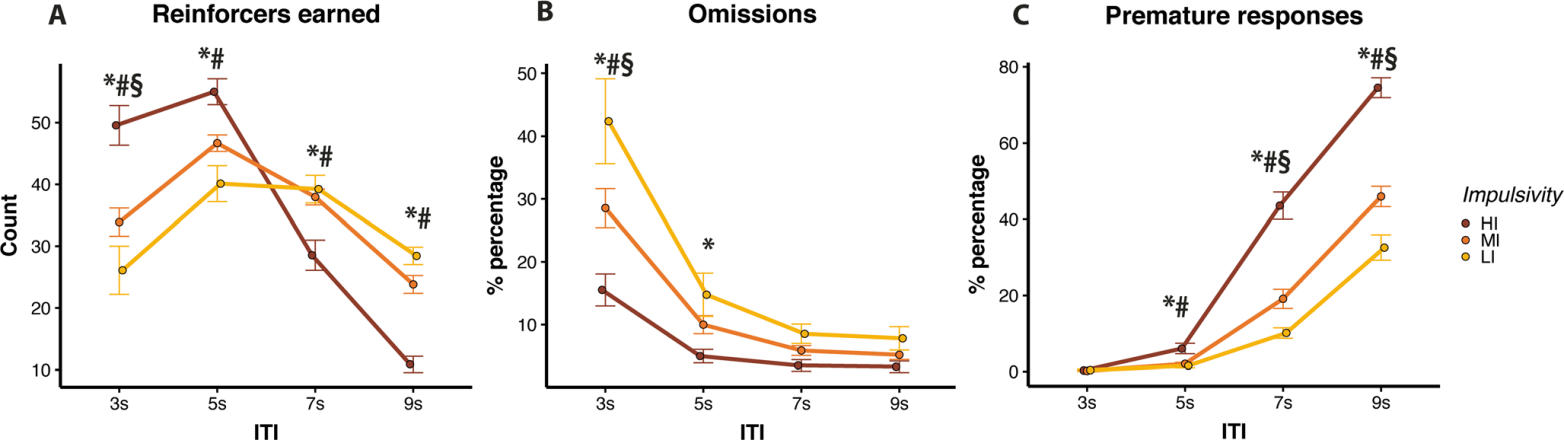
Trait impulsivity modulates performance on a vITI paradigm on 5CSRTT. Group differences for Day 2 in (**A**) reinforcers earned, (**B**) percentages of omission errors and (**C**) percentages of premature responses. *HI vs LI *p* < 0.05; #HI vs MI *p* < 0.05; §MI vs LI *p* < 0.05

For reinforcers earned, there was a significant Day × ITI × Group interaction (*F*(6,231) = 2.89, *p* = 0.010). Since the three-way interaction was significant, separate multilevel models were used to ascertain the Group dependency of the ITI effects in each Day separately. Impulsivity phenotype determined the efficacy of performance in terms of earned reinforcers at different ITI values for the second day of testing (Group × ITI interaction, *F*(6,99) = 21.91, *p* < 0.001). This is shown in Fig. 1A where HI rats obtained more reinforcers than LI rats (*t* = 6.30, *p* < 0.001) and MI (*t* = 4.51, *p* < 0.001) on the short 3 s ITI trials, with MI rats also earning more reinforcers than LI on the short 3 s ITI trials (*t* = 2.77, *p* = 0.018). HI also earned more reinforcers than LI on the 5 s ITI trials (*t* = 2.81, *p* = 0.002) but earned fewer reinforcers than LI (*t* = −3.65, *p* = 0.012; *t* = −4.69, *p* < 0.001) and MI (*t* = −2.87, *p* = 0.009; *t* = −2.55, *p* < 0.001) on the long 7 s and 9 s ITI trials, respectively. A similar effect was evident on day 1 of testing as shown by Figure S1a in the supplementary materials. In summary, HI rats earned more reinforcers at shorter ITI trials, whilst LI rats earned more reinforcers at longer ITI trials.

Both impulsivity phenotype and ITI influenced the incidence of omission responses (Group × ITI, *F*(6,231) = 2.76, *p* = 0.013). Figure 1B shows data for the second day of testing. LI rats made proportionally more omission responses than HI rats (*t* = −5.06, *p* < 0.001) and MI (*t* = −3.82, *p* <0.001) on the short 3 s ITI. LI rats also made more percentages of omission errors than HI rats on the 5 s ITI trials (*t* = −2.80, *p* = 0.018). In summary, LI rats were more prone than HI and MI at making omission errors, and these occurred on short ITI trials.

For percentages of premature responses, there was a significant Day × ITI × Group interaction (*F*(6,231) = 3.26, *p* = 0.004). Since the three-way interaction was significant, separate multilevel models were used to ascertain the Group dependency of the ITI effects in each Day separately. Impulsivity phenotype and ITI influenced the frequency of premature responses in the second day of testing (Group × ITI interaction, *F*(6,99) = 12.92, *p* < 0.001). Figure 1C shows that HI rats made proportionally more premature response than LI and MI rats during 5 s ITIs (*t* = 3.34, *p* = .003; *t* = 3.77, *p* < 0.001, respectively), 7 s ITIs (*t* = 9.16, *p* < 0.001; *t* = 7.24, *p* < 0.001, respectively) and 9 s ITIs (*t* = 7.96, *p* < .001; *t* = 5.86, *p* < 0.001, respectively). MI rats also made more percentages of premature response than LI rats on the 7 s ITI (*t* = 3.34, *p* = 0.003) and the 9 s ITI (*t* = 3.33, *p* = 0.004). A similar pattern was evident on day 1 as shown by Figure S1b in the supplementary materials. In summary, HI rats and to an extent MI rats made proportionally more premature responses than LI rats, and these occurred during the long ITI trials.

We then combined premature, correct and incorrect responses and divided the session into 5-min bins to examine whether HI, MI and LI rats differ in overall rate of responding. There was an effect of impulsivity phenotype on number of active responses per unit of time (*F*(2,33) = 6.32, *p* = 0.005). Specifically, HI rats were significantly more active than LI rats (*t* = 3.54, *p* = 0.003). For more details on this, see Figure S2 in the supplementary materials.

We next assessed the relationships between the various behavioural variables. During the first (*r* = −0.46, *p* = 0.005) and second (*r* = −0.37, *p* = 0.028) day of testing, there was an overall significant negative relationship between making an omission on the 3 s ITI and making a premature response on the 9 s ITI. There was also a strong positive correlation between making a correct response on the 3 s ITI and making a premature response on the 9 s ITI both on day 1 (*r* = 0.64, *p* < 0.001) and on day 2 (*r* = 0.64, *p* < 0.001). These correlations are in line with behavioural data analysed by impulsivity phenotype, showing that animals that respond prematurely during long ITI trials are also more likely to respond correctly on short ITI trials. Conversely, animals that do not engage with rapid, short ITI trials, and thus make many omissions on these trials, are more likely to respond correctly when waiting is rewarded. Finally, impulsivity groups and ITI types influenced latency to perform correct and premature responses. For more details on this, see Table 1.

**Table 1:** Experiment 1, vITI challenge. Latencies for correct, incorrect and premature responses.

	**Correct responses**				**Incorrect responses**				**Premature responses**	
	**3 s**	**5 s**	**7 s**	**9 s**	**3 s**	**5 s**	**7 s**	**9 s**	**7 s**	**9 s**
HI	937.2±82.4*	643.1±22.2*	596.5±19.7*	660.3±53.6*	2545.9±140	1651±123.6	1014.4±106.8	1113.2±173.5	5674.8 ± 53°*	6479.4 ± 79.3°*
MI	1101.7±60.3	744.4±25.5	620.5±18.8	680.2±27.9	2768.1±105.7	1789.6±107.9	1445.6±110.5	1197.1±149.4	**5946.5±54.2°**	**6901.1±66.6°**
LI	1324.4±94.5*	901±51.1*	758.2±25.1*	709.9±27.4*	2712.2±151.5	2176.1±117.8	1301.6±124.2	1347.6±217	5766.8±85.3*	7129.9±69.6*

*HI vs LI *p* < 0.05

°HI vs MI *p* < 0.05

#### Short vITI challenge

**Fig. 2:**
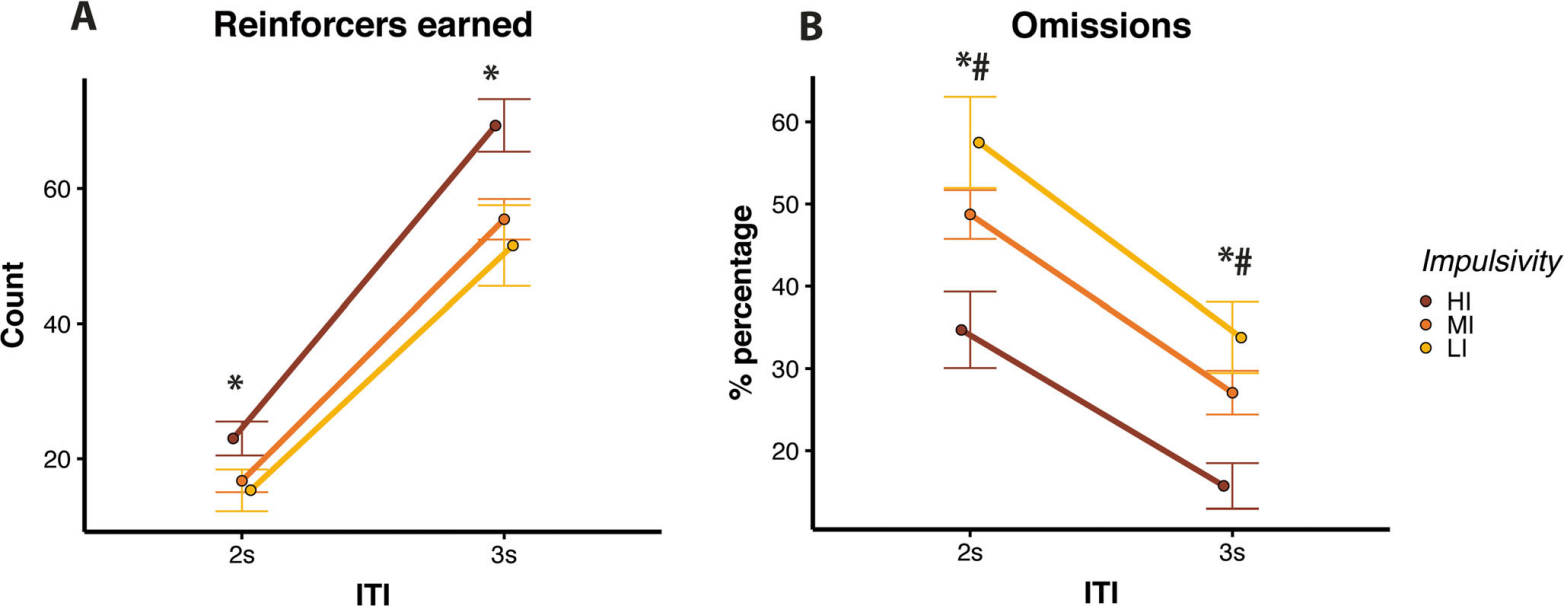
Trait impulsivity modulates performance on a short vITI paradigm on 5CSRTT. Group differences in (**A**) reinforcers earned and (**B**) percentages of omission errors. *HI vs LI *p* < 0.05; #HI vs MI *p* < 0.05

We next tested rats with short vITI trials of 2 and 3 s (mean of the vITI session: 2.6 s). Figure 2A shows that the impulsivity groups differed with regard to reinforcers earned (*F*(2,33) = 3.65, *p* = 0.037) with HI rats earning significantly more reinforcers than LI rats (*t* = 2.53, *p* = 0.041). As shown in Fig. 2B, omissions varied as a function of ITI (*F*(1,33) = 206.33, *p* < 0.001) and impulsivity group (*F*(2,33) = 7.53, *p* = 0.002). Percentages of omission errors were higher during 2 s ITI trials than 3 s ITI trials (*t* = 14.36, *p* < .001), and HI rats made significantly less of these errors than LI (*t* = −3.77, *p* = 0.002) and MI (*t* = −2.94, *p* = 0.023) rats. These findings show that HI rats show a superior performance compared with LI rats during fast-paced trials.

Latencies to make a correct response varied significantly across different ITIs (*F*(1,33) = 69.29, *p* < 0.001) and impulsivity groups (*F*(2,33) = 4.17, *p* = 0.024). Latencies to make an incorrect response also varied depending on ITI (*F*(1,33) = 6.52, *p* = 0.015). For details on this, see Table S1 in the supplementary materials.

### Experiment 2: Effects of methylphenidate, atomoxetine, amphetamine, atipamezole and phenylephrine on vITI performance

Prior to the drug administration studies, rats were trained to a stable baseline level and were tested on three vITI sessions. On baseline, HI rats exhibited elevated premature responses and lower accuracy compared with LI rats (for more details, see the supplementary materials).

Results from the vITI challenges replicated Experiment 1 and are shown in the supplementary section (see Table S2 and Figures S3–S5). Briefly, HI rats earned more rewards and made fewer omissions when the task required rapid information in short ITI trials. When the ITI was increased to longer durations, HI rats showed more premature responses than LI rats. LI rats exhibited the opposite behavioural profile with more rewards during long ITI trials and impaired performance during the short ITI trials with increased omissions. Finally, rats were also tested on a fixed 7 s ITI session, to allow comparisons with previous publications. Briefly, HI rats categorised using the variable ITI procedure exhibited significantly increased levels of premature responding compared with LI rats and MI rats during a fixed 7 s ITI session (for more details see, the supplementary materials).

**Fig. 3:**
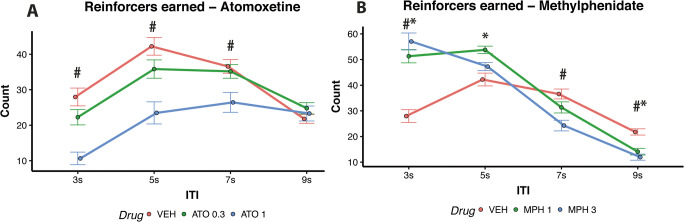
(**A**) Effects of ATO on reinforcers earned; *low-dose vs vehicle *p* < 0.05; #high-dose vs vehicle *p* < 0.05. (**B**) Effects of MPH on reinforcers earned; *low-dose vs vehicle *p* < 0.05; #high-dose vs vehicle *p* < 0.05

Figure 3A,B shows that the effects of ATO and MPH on behaviour depended on the ITI (Drug × ITI interaction, *F*(12,380) = 28.52, *p* < 0.001). As shown in Fig. 3A, during short 3 s and 5 s ITI trials, ATO (1 mg/kg) significantly decreased the number of reinforcers earned compared with vehicle (*t* = 7.23, *p* < .001; *t* = 6.39, *p* < .001 respectively for 3 s and 5 s ITI trials). ATO (1 mg/kg) also reduced the number of reinforcers earned during the long 7 s ITIs compared with the vehicle group (*t* = 3.89, *p* < 0.001).

In contrast, during short 3 s ITI trials, rats earned more reinforcers following the administration of MPH at both low (1 mg/kg, *t* = 6.91, *p* < 0.001) and high (3 mg/kg, *t* = 7.92, *p* < 0.001) doses compared with the vehicle group. The beneficial effect of 1 mg/kg MPH extended to the 5 s ITI compared to vehicle (*t* = 3.20, *p* = 0.005). However, similar to ATO, during long ITI trials with high-dose MPH (3 mg/kg), performance deteriorated both on the 7 s and 9 s ITI trials (*t* = 4.10, *p* < 0.001; *t* = 4.41, *p* < 0.001 respectively). Low-dose MPH (1 mg/kg) impaired performance during the 9 s ITI trials (*t* = 3.49, *p* = 0.002).

ATO affected performance differently depending on the impulsivity phenotype (Drug × Group interaction, *F*(8,380) = 2.31, *p* = 0.020). Specifically, high-dose ATO (1 mg/kg) worsened performance mostly of MI (*t* = 5.72, *p* < 0.001) and LI rats (*t* = 7.66, *p* < 0.001) and only produced a trend level decrement in performance for HI rats (*t* = 2.37, *p* = 0.063, see Fig. 6A).

**Fig. 4:**
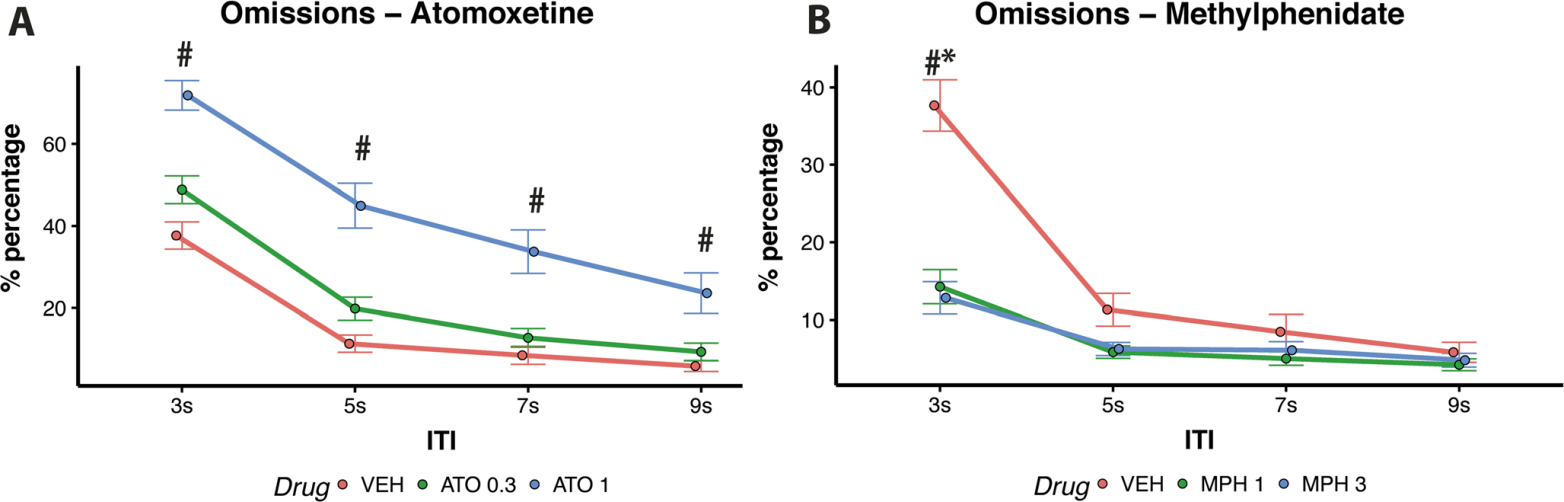
(**A**) Effects of ATO on percentages of omission errors; *low-dose vs vehicle *p* < 0.05; #high-dose vs vehicle *p* < 0.05. (**B**) Effects of MPH on percentages of omission errors; *low-dose vs vehicle *p* < 0.05; #high-dose vs vehicle *p* < 0.05

ATO and MPH affected the percentages of omission errors differently depending on the ITI (Drug × ITI, *F*(12,380) = 5.38, *p* < 0.001). Figure 4A shows that treatment with high-dose ATO (1 mg/kg) increased the percentages of omission responses compared to vehicle on all ITIs (3 s ITI: *t* = 6.82, *p* < 0.001; 5 s ITI: *t* = 8.43, *p* < 0.001; 7 s ITI: *t* = 7.17, *p* < 0.001; 9 s ITI: *t* = 5.78, *p* < 0.001).

In contrast, Fig. 4B shows that treatment with both high (3 mg/kg) and low-dose (1 mg/kg) MPH reduced the percentages of omission responses on short 3 s ITI trials (*t* = −7.16, *p* < 0.001; *t* = −6.82, *p* < 0.001 for the high and low doses respectively).

Both ATO and MPH modulated performance differently depending on the impulsivity phenotype (Group × Drug, *F*(8,380) = 2.20, *p* = 0.026). Specifically, low-dose ATO (0.3 mg/kg) increased the percentage of omission responses solely for MI rats (*t* = 4.74, *p* < 0.001) whilst high-dose (3 mg/kg) and low-dose (1 mg/kg) MPH decreased the percentage of omission responses both for HI (*t* = 4.42, *p* = 0.001; *t* = 4.05, *p* < 0.001; respectively) and LI rats (*t* = 3.45, *p* = 0.004; *t* = 3.86, *p* = 0.013; respectively; see Fig. 6C,D).

**Fig. 5:**
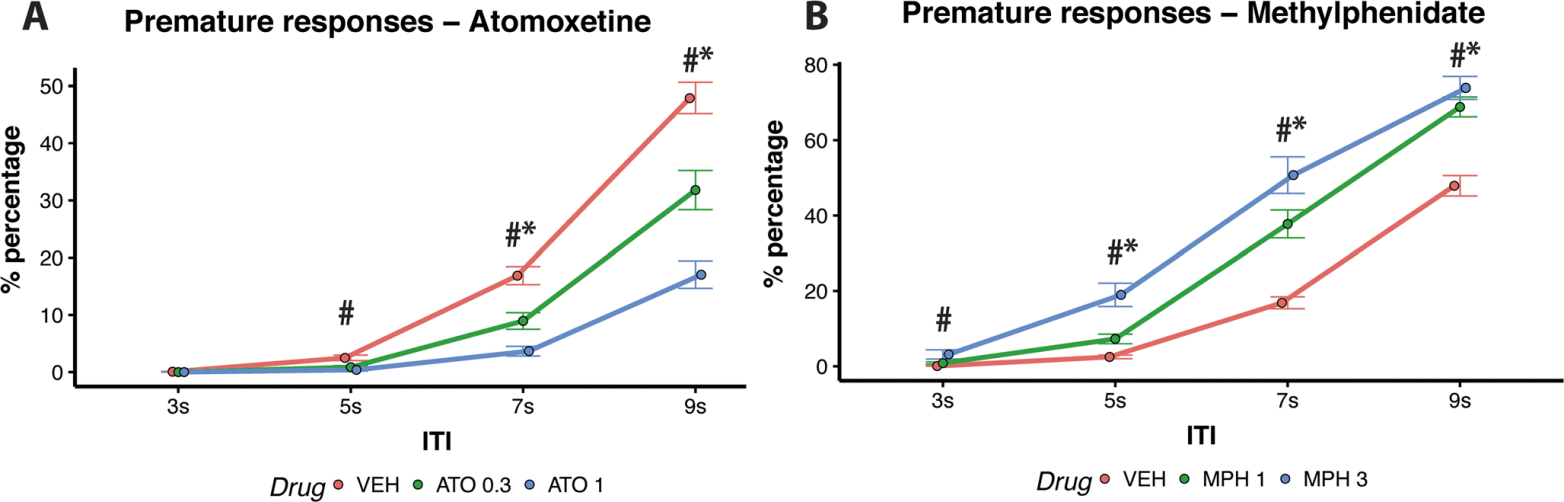
(**A**) Effects of ATO on percentages of premature responses; *low-dose vs vehicle *p* < 0.05; #high-dose vs vehicle *p* < 0.05. (**B**) Effects of MPH on percentages of premature responses; *low-dose vs vehicle *p* < 0.05; #high-dose vs vehicle *p* < 0.05

ATO and MPH affected the percentages of premature response differently depending on the ITI (Drug × ITI, *F*(12,380) = 15.02, *p* < 0.001). Figure 5A shows that administration of ATO both low-dose (0.3 mg/kg) and high-dose (1 mg/kg) decreased the percentage of premature responses on trials with 7 s (*t* = 3.75, *p* < 0.01; *t* = 7.62, *p* < 0.01 for the low and high doses respectively) and 9 s ITIs (*t* = 3.74, *p* < 0.01; *t* = 9.44, *p* < 0.01 for the low and high doses respectively). High-dose ATO (1 mg/kg) also decreased the percentage of premature responses on the 5 s ITI trials (*t* = 3.40, *p* = 0.003). On the contrary, Fig. 5B shows that administration of both low-dose (1 mg/kg) and high-dose (3 mg/kg) MPH increased the percentage of premature responses on the 5 s, 7 s and 9 s ITI trials (low-dose: 5 s ITI *t* = 3.73, *p* < 0.001; 7 s ITI *t* = 6.96, *p* < 0.001; 9 s ITI *t* = 4.86, *p* < 0.001; high-dose: 5 s ITI *t* = 9.07, *p* < 0.001; 7 s ITI *t* = 9.99, *p* < 0.001; 9 s ITI *t* = 5.86, *p* < 0.001). High-dose MPH (3 mg/kg) also increased the percentage of premature responses in the 3 s ITI trials (*t* = 3.71, *p* < 0.001).

**Fig. 6:**
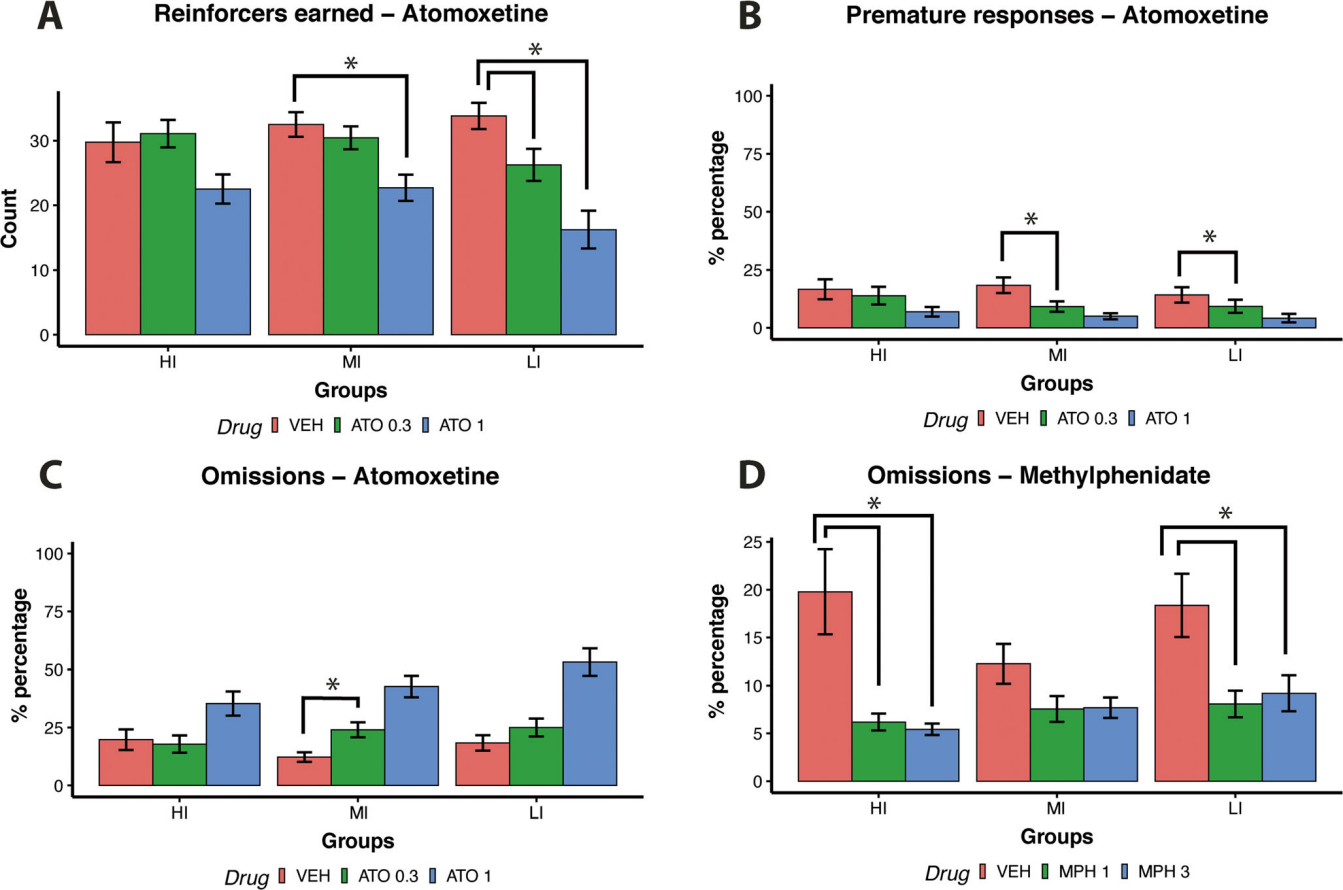
(**A–D**) Interaction between drug and trait impulsivity. Effects of ATO and MPH on reinforcers earned, percentages of premature responses and percentages of omissions. *p < 0.05

ATO modulated performance differently depending on impulsivity phenotype (Group × Drug, *F*(8,380) = 2.82, *p* = 0.004). Specifically, low-dose ATO (0.3 mg/kg) decreased the percentage of premature responses compared with vehicle in MI (*t* = 5.38, *p* < 0.001) and LI (*t* = 2.98, *p* = 0.012) but not in HI rats (*t* = 0.83, *p* = 0.780; see Fig. 6B). Latencies on correct, incorrect and premature responses following administration of ATO and MPH are shown in Tables S3–S5 of the supplementary materials.

The above findings show that MPH and ATO have essentially opposite effects on performance. Whereas MPH led to a general activation of behaviour, increasing premature responses, decreasing omissions and facilitating responding on short ITI trials, the administration of ATO produced a general inhibition of behaviour with reduced premature responses during long ITI trials and increasing omissions, especially during short ITI trials. Finally, the action of ATO was dependent on trait impulsivity and affected MI and LI rats more so than HI rats.

#### Effects of amphetamine, atipamezole and phenylephrine

**Fig. 7:**
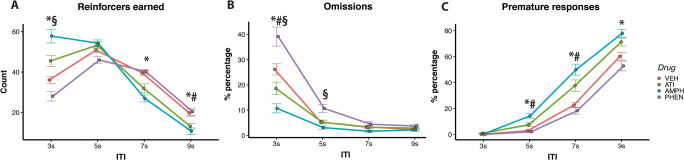
Effects of AMPH, ATI and PHEN on (**A**) reinforcers earned, (**B**) percentages of omission responses and (**C**) percentages of premature responses *AMPH vs vehicle *p* < 0.05; #ATI vs vehicle *p* < 0.05; §PHEN vs vehicle *p* < 0.05

Figure 7A shows that AMPH, PHEN and ATI affected performance on long and short ITI trials differently depending on the ITI (Drug × ITI, *F*(9,285) = 17.19, *p* < 0.001). Specifically, in the short 3 s ITI trials, animals earned more pellets after administration of AMPH compared with vehicle (*t* = 5.62, *p* < 0.001), but less pellets after administration of PHEN (*t* = −3.42, *p* = 0.002). There was also a trend for animals to earn more pellets on 3 s ITI trials following the administration of ATI compared to vehicle (*t* = 2.25, *p* = 0.068). During long 7 s and 9 s ITI trials, animals earned significantly fewer pellets following administration of AMPH compared to vehicle (*t* = −4.28, *p* < 0.001; *t* = −4.69, *p* < 0.001 respectively). During 7 s ITI trials, there was a trend for animals to earn less pellets following administration of ATI compared to vehicle (*t* = −2.27, *p* = 0.064). This effect was significant in the 9 s ITI trials (*t* = −3.20, *p* = 0.004).

Omission responses were affected differently by AMPH, ATI and PHEN depending on the ITI (Drug × ITI, *F*(9,285) = 5.88, *p* < 0.001). Figure 7B shows that treatment with ATI (*t* = 2.30, *p* = 0.05) and AMPH (*t* = 6.10, *p* < .001) reduced the percentage of omission responses on the short 3 s ITI trials; however, PHEN increased the percentage of omission responses on the 3 s and 5 s ITI trials (*t* = 4.49, *p* < 0.001, *t* = 3.69, *p* = 0.001 respectively). In addition, AMPH reduced omission responses on the 7 s ITI trials (*t* = 2.41, *p* = 0.046).

Premature responses were affected differently by AMPH, ATI and PHEN depending on the ITI (Drug × ITI, *F*(9,285) = 4.77, *p* < 0.001). Figure 7C shows administration of AMPH increased the percentage of premature responses in trials with 5 s (*t* = 6.07, *p* < .001), 7 s (*t* = 6.76, *p* < .001), 9 s (*t* = 3.15, *p* < .001) ITIs, whereas ATI only increased premature responses during 5 s (*t* = 2.83, *p* = 0.014) and 7 s (*t* = 3.08, *p* = 0.007) ITIs. Latencies on correct, incorrect and premature responses following administration of ATI, AMPH and PHEN are shown in Table S6 of the supplementary materials.

The effects of AMPH, ATI and PHEN were determined by ITI. Administration of AMPH and ATI led to behavioural disinhibition: increasing premature responses, decreasing omissions and facilitating responding on fast-paced, short ITI trials. Administration of PHEN, instead, led to a general inhibition of behaviour: reducing premature responses and increasing omissions, especially during short ITI trials.

## Discussion

The findings show that high and low levels of impulsivity can be both detrimental and advantageous to task performance, depending on the precise contingencies of the test environment. Specifically, HI rats performed best with short ITIs and fast stimulus presentations whilst LI rats were superior during the long ITI trials of the vITI-5CSRTT. Moreover, the effects of drugs used to treat ADHD also depended on the context of the test situation as well as baseline levels of impulsivity. Specifically, drugs that increase the levels of catecholamines both cortically and subcortically, such as MPH and AMPH, conferred an advantage in the short ITI trials of the 5CSRTT and mimicked the behavioural profile of HI rats. In contrast, the selective NA reuptake blocker ATO (Swanson et al. 2006) which also increases DA in the prefrontal cortex but has no effect on DA levels in the striatum (Carboni et al. 2006) decreased impulsivity, slowed response latencies and improved performance in long ITI trials, mimicking the behaviour of LI rats. Importantly, we noticed that the effect of ATO was partly dependent on trait impulsivity and exerted greater influence on behaviour in MI and LI rats. The contrasting effects of MPH and ATO implicate DA and NA in different aspects of sustained attentional performance.

In two separate experiments, over multiple sessions, HI animals earned more reinforcers and made fewer omission responses than LI and MI animals in the short, 3 s and 5 s ITI trials. In addition, HI were on average faster at making a correct response, regardless of ITI. This adaptive response was not the result of a strategy chosen based on the available contingencies. Indeed, when animals were challenged with a session that presented only short ITIs, LI (and to a lesser extent MI) rats were not able to adapt to the short latencies and performed significantly worse than HI rats. Conversely, when the task required animals to wait for an extended period before responding, the behavioural phenotype typical of HI rats emerged, with increased premature responses during the long 7 s and 9 s ITIs. This suggests that there is some adaptive value to the impulsivity phenotype. The advantage that this trait confers is revealed under high-event rate conditions, where rapid information processing, including visual attention and action, is required. The significance of these findings can be seen from work in humans showing that task pace and frequency of reinforcers improve performance of impulsive subjects (Cools et al. 2005) and ADHD juveniles (Delisle and Braun 2011; McInerney and Kerns 2003; Slusarek et al. 2001; Strand et al. 2012), normalising behaviour or even improving behaviour compared with matched healthy controls. Some have attributed this phenomenon to an intolerance of delayed rewards and increased susceptibility to boredom (Barkley 2001; Wiersema et al. 2006), suggesting that uninteresting or non-stimulating tasks foster the development of ADHD symptoms, whilst fast-paced and motivating contexts reduce their occurrence. This is particularly relevant for the present study with a superior performance of HI rats during a high-event rate challenge.

LI rats tended to stop responding on the fast trials but continued to work on the 7 s and 9 s ITI trials. Indeed, HI rats were on average faster and more active than LI rats throughout the session. Short latencies and greater activity-per-unit-of-time have been postulated to require greater energy and thus be more costly (Niv et al. 2005, 2007; Opris et al. 2011; Staddon 2001). Niv et al. (2005, 2007) explored this idea computationally, suggesting that the expectation of future reward determines the rapidity or vigour with which the operant is performed by functioning as an opportunity cost, that is by determining whether the cost of responding fast and/or frequently is worth the outcome. Building on this idea, both rats (Opris et al. 2011) and humans (Shadmehr 2010) respond faster and with greater vigour when the opportunity to gain a reward is high, whereas they are slower and less active when the expected reward from any given action is low. This corroborates the hypothesis that movement kinematics are dictated by the value attributed to a stimulus and by the rate at which this value is discounted in time (Shadmehr 2010). This would suggest that steeper than normal temporal discounting of reward would be accompanied by faster and more vigorous movements. Consistent with this idea, subjects showing impaired waiting impulsivity (Choi et al. 2014; Wallace and Newman 1990) or proneness to boredom (Berret et al. 2018) make faster and more vigorous movements.

This is in line with our data, showing that high impulsive animals, who are steeper discounters (Robinson et al. 2009), have faster responding and demonstrate superior performance when such behaviour is advantageous. According to the normative account described by Niv et al. (2007), the more vigorous responding of HI rats would indicate enhanced subjective utility of food reward for this endophenotype. Interestingly, both in rodents and in humans, impulsive action has been associated with indices of greater sensitivity to reward-predicting cues, such as risky decision-making (Barrus et al. 2015; Gabriel et al. 2019; Ioannidis et al. 2019), substance abuse (Belin et al. 2008; Dalley et al. 2007; Diergaarde et al. 2008, 2009; Voon 2014) and increased responsivity for sucrose (Diergaarde et al. 2009). Recent data in humans supports the idea that motor impulsivity is associated with enhanced value attribution to reinforcers (Mechelmans et al. 2017); however, we did not test this directly in this study.

Results from the pharmacological interventions show that the effects that drugs have on behavioural performance can be both context and trait dependent. Specifically, drugs that increase the release of dopamine and noradrenaline both cortically and subcortically, such as MPH and AMPH (Bymaster et al. 2002; Kuczenski and Segal 2001), reduced response latencies and improved performance on fast-paced trials. In long ITI trials, instead, they increased premature responses and deteriorated performance, mimicking, as a whole, the behaviour of HI rats. Behavioural results obtained with these drugs agree with previous research on impulsive action, whereby increasing extracellular DA levels, in particular in the striatum (Economidou et al. 2012), leads to enhanced behavioural activation and more premature responses (Baarendse and Vanderschuren 2012; Milstein et al. 2010; Murphy et al. 2008; Navarra et al. 2008; Pattij et al. 2007; Sun et al. 2012). Navarra et al. (2008) had also observed an improvement of performance on the 5CSRTT with MPH on some short ITI trials; we confirm these findings and extend this effect to low-dose AMPH. These results add to a growing body of literature on the pro-cognitive effects of psychostimulants in animals (Paine et al. 2007; Tomlinson et al. 2014; Turner et al. 2016) and in humans (Pietrzak et al. 2006; Wardle et al. 2011). These results are also in line with computational (Niv et al. 2005, 2007) and experimental evidence (Hamid et al. 2016; Klaus et al. 2019; Mohebi et al. 2019; Wassum et al. 2012) that increased DA transmission in the striatum lowers the threshold for action initiation and invigorates operant responding by a process of activation (Robbins and Everitt 2007). Importantly, these findings also suggest that the advantage conferred by impulsivity in highly stimulating contexts may be due to increased levels of dopamine in the striatum. This is in line with evidence suggesting that HI rats present increased synaptic DA levels in the shell sub-region of the ventral striatum due to reduced expression of the dopamine transporter and decreased DA D2/D3 receptor availability in this region (Dalley and Robbins 2017; Jupp et al. 2013).

Contrary to MPH, systemic administration of ATO, which increases extracellular NA levels and does not affect DA release in the striatum but increases it in the PFC (Carboni et al. 2006; Swanson et al. 2006), improved performance on long ITI trials, increased omissions on short ITI trials and slowed responding in general, mimicking the behaviour of LI rats. These results are in line with previous research on 5CSRTT showing that ATO reduces premature responses in this task (Blondeau and Dellu-Hagedorn 2007; Economidou et al. 2012; Fernando et al. 2012; Navarra et al. 2008; Robinson et al. 2008). Contrary to previous evidence (Navarra et al. 2008 5 s ITI 1.0mg/kg; Callahan et al. 2019 2.5 s ITI 3.0mg/kg), however, we did not observe an increase in the probability to make a correct response in short ITI trials following administration of ATO, and instead we saw a decrement in performance, consistent with a role of ATO in reducing behavioural activation or even producing mild sedative effects. Importantly, MI and LI rats were more sensitive to the deactivating effects of ATO than HI rats; thus, trait-related factors can determine the behavioural effects of ATO. Studies using tasks other than the 5CSRTT have also observed reduced behavioural responding following the administration of ATO, such as an increase in omissions on a cognitive-effort task (Hosking et al. 2015) and a decrease in breakpoint in a progressive ratio choice task (Yohn et al. 2016). The specific mechanisms of how ATO strengthens behavioural inhibition are not well understood. In the context of 5CSRTT, it is possible that ATO reduces premature responses by increasing NA transmission in the nucleus accumbens shell (Benn and Robinson 2017; Economidou et al. 2012). Others have found that administration of ATO reduces DA release in the nucleus accumbens core, with a concomitant behavioural effect of decreased willingness to exert effort in a progressive ratio task (Yohn et al. 2016). The authors then speculated that ATO may be acting via alpha-2 adrenergic receptors on dopamine neurons of the ventral tegmental area (VTA) to reduce accumbal DA release (Guiard et al. 2008; Yohn et al. 2016).

Blockade of the alpha-2a adrenoceptors with ATI yielded results similar to those of psychostimulants, with a decrease of omissions in short ITI trials and an increase in premature responses in long ITI trials. In contrast, the alpha1 adrenoceptor agonist PHEN resulted in a behavioural profile similar to that of ATO, reducing correct responses and increasing the probability of omissions in short ITI trials. These results are in line with previous research in 5CSRTT (Koskinen et al. 2003; Pattij et al. 2012; Sirviö et al. 1993) showing that PHEN leads to an overall inhibition of responding whilst administration of ATI results in an increase in behavioural activation. Given that administration of ATI yielded results markedly different from those of ATO, it is unlikely that ATI is acting on pre-synaptic alpha-2a autoreceptors on NA fibres to activate NA transmission (Berridge and Waterhouse 2003). Instead, ATI may be acting on post-synaptic alpha-2a adrenoceptors subcortically to increase DA release, either via its action on alpha-2-autoreceptors on VTA DA cells (Guiard et al. 2008), or by inhibiting DA decline in the striatum (Yavich et al. 2003). Alternatively, ATI may act on post-synaptic alpha-2a adrenoceptors receptors located in the prefrontal cortex. Studies in non-human primates have shown that blockade of these receptors with infusions of yohimbine, an alpha-2a antagonist, in the dorsolateral prefrontal cortex, increases impulsivity (Ma et al. 2003) and induces locomotor hyperactivity (Ma et al. 2005).

NA has lower affinity for alpha-1 adrenoceptors compared to alpha-2 adrenoceptors (Mohell et al. 1983; O’Rourke et al. 1994), and thus alpha-1 adrenoceptors in the prefrontal cortex are thought to be preferentially engaged during high levels of stress, when levels of NA release are highest (Ramos and Arnsten 2007). There is evidence that activation of alpha-1 adrenoceptors in prefrontal cortex impairs working memory performance both in rodents (Arnsten et al. 1999; Birnbaum et al. 2004) and monkeys (Arnsten and Jentsch 1997; Birnbaum et al. 2004; Mao et al. 1999). On the basis of this, it is possible that the slowing of behavioural activation that we observe with PHEN could be due to a neocortical action of this drug.

In conclusion, our findings demonstrate that trait impulsivity can be advantageous in certain contexts, specifically when rapid responding and attentional focusing is required. From human studies on ADHD, it is apparent that stimulating environments can help remediate decrements in performance in this patient population; however, we have demonstrated, for the first time to our knowledge, that this is also true in animal models of impulsivity. Importantly, we have also explored the role that catecholamines play in the performance of high-event rate tasks, and we suggest that drugs that elevate subcortical*,* as well as cortical, DA levels improve performance on fast-paced trials, whilst drugs that act mainly to block the reuptake of NA slow responding in such situations. These results have important implications for our understanding of impulsivity, the context within which it manifests and the pharmacological agents that are used to treat ADHD.

## Supplementary Information

Figure S1 (PNG 321 kb)

High resolution image (TIF 34340 kb)

Figure S2 (PNG 460 kb)

High resolution image (TIF 29963 kb)

Figure S3 (PNG 322 kb)

High resolution image (TIF 34252 kb)

Figure S4 (PNG 207 kb)

High resolution image (TIF 29239 kb)

Figure S5 (PNG 605 kb)

High resolution image (TIF 37388 kb)

Figure S6 (PNG 566 kb)

High resolution image (TIF 49003 kb)

Figure S7 (PNG 473 kb)

High resolution image (TIF 49621 kb)

ESM 1 (DOCX 31 kb)
