# Supplementary figures and images for: Adaptive aspects of impulsivity and interactions with effects of catecholaminergic agents in the 5-choice serial reaction time task: implications for ADHD
Source: Psychopharmacology (Berl). 2021 Jun 9;238(9):2601–15. doi: 10.1007/s00213-021-05883-y (PMC8373759; doi:10.1007/s00213-021-05883-y)

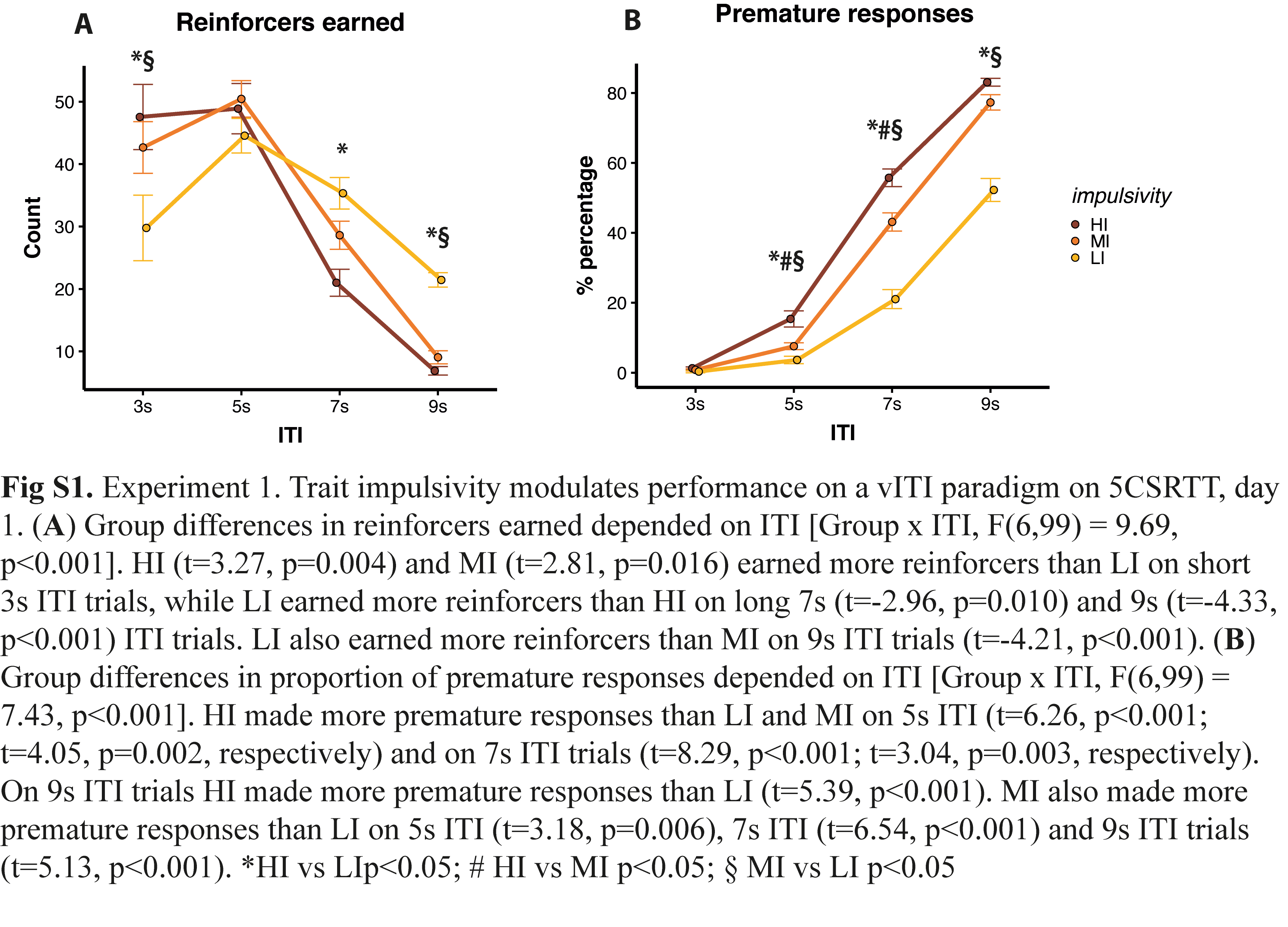

Supplement: Supplementary file 1 — (PNG 321 kb) [file 213_2021_5883_Fig1_ESM.png]

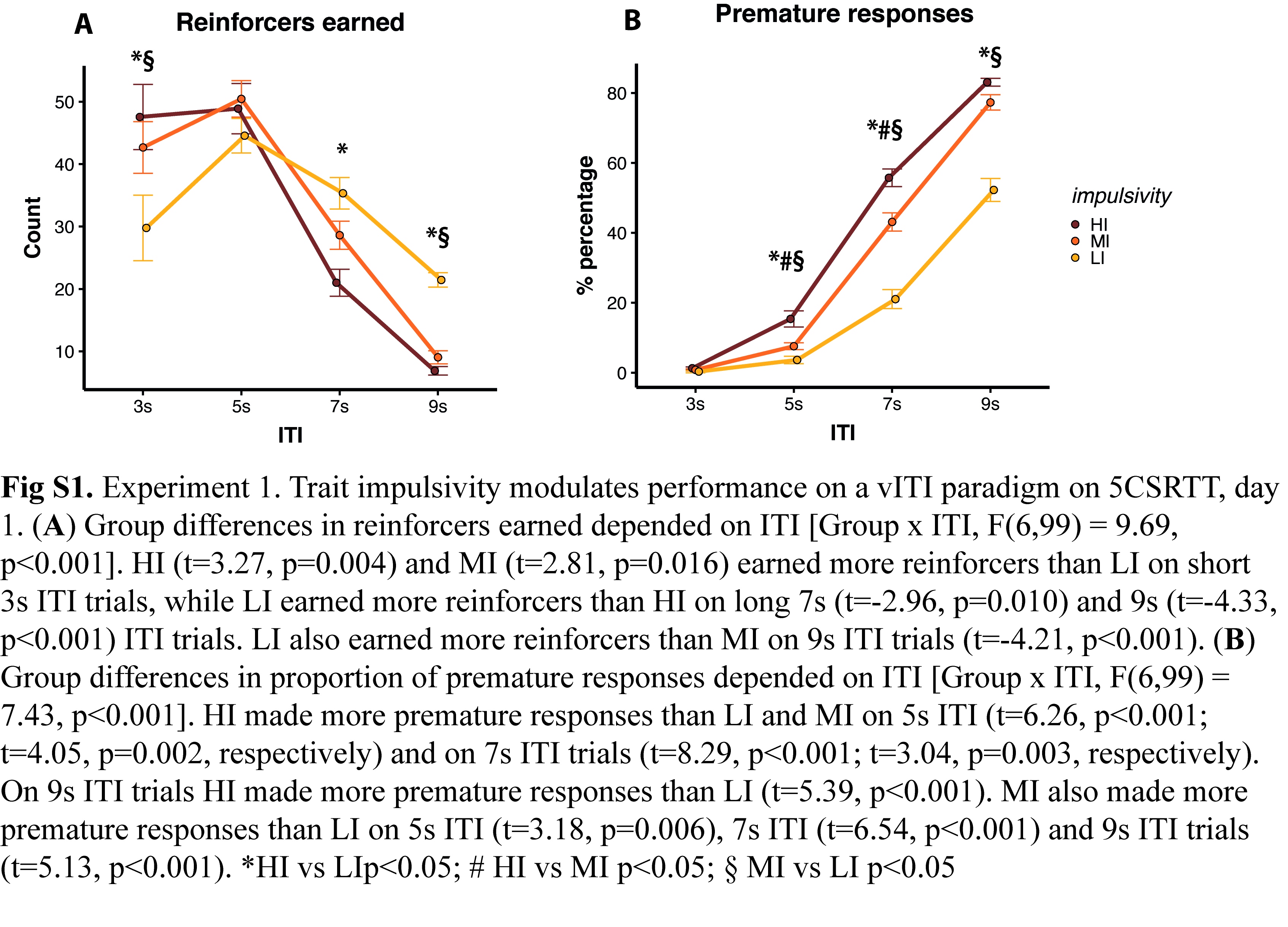

Supplement: Supplementary file 2 — High resolution image (TIF 34340 kb) [file 213_2021_5883_MOESM1_ESM.tif]

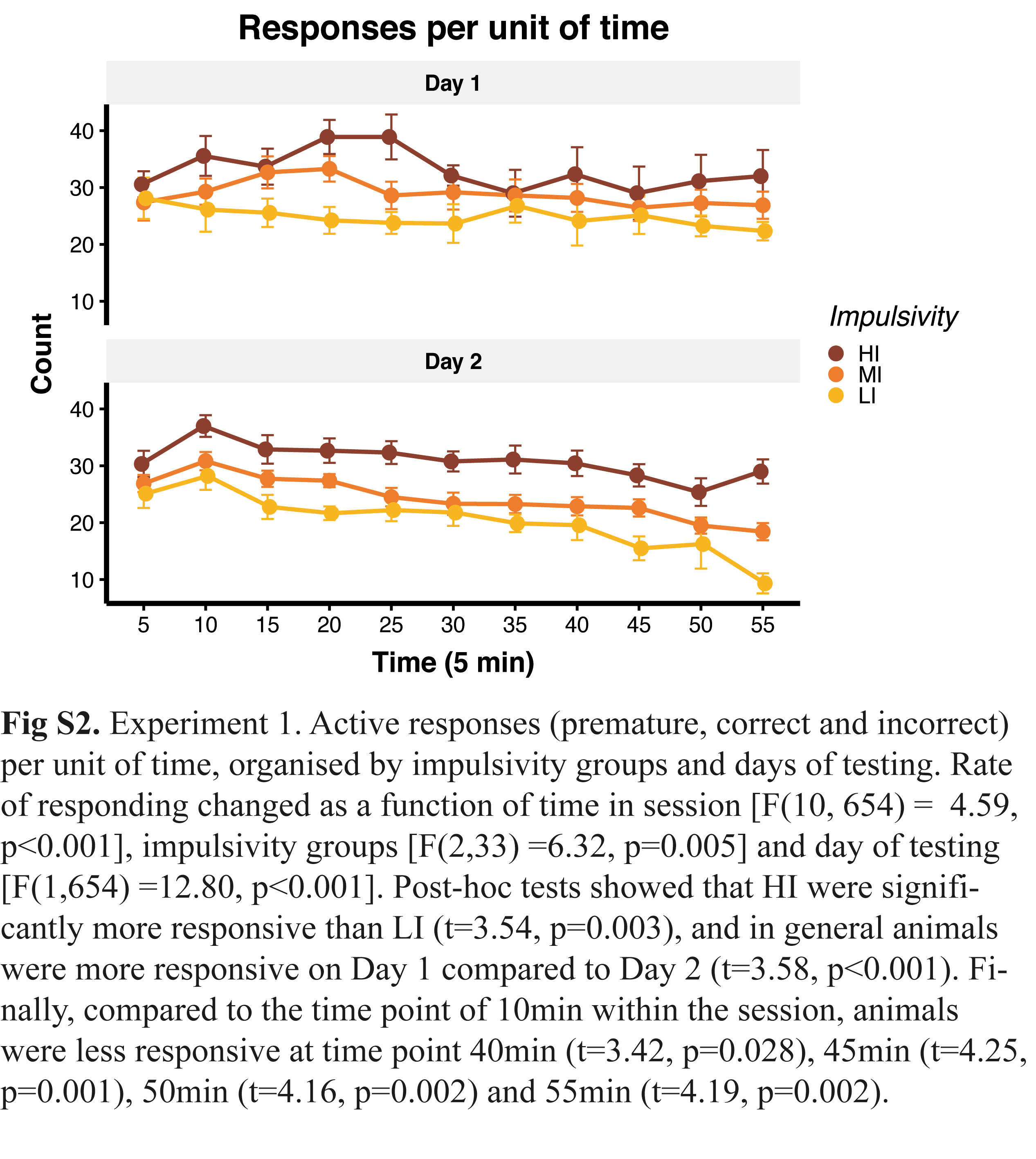

Supplement: Supplementary file 3 — (PNG 460 kb) [file 213_2021_5883_Fig2_ESM.png]

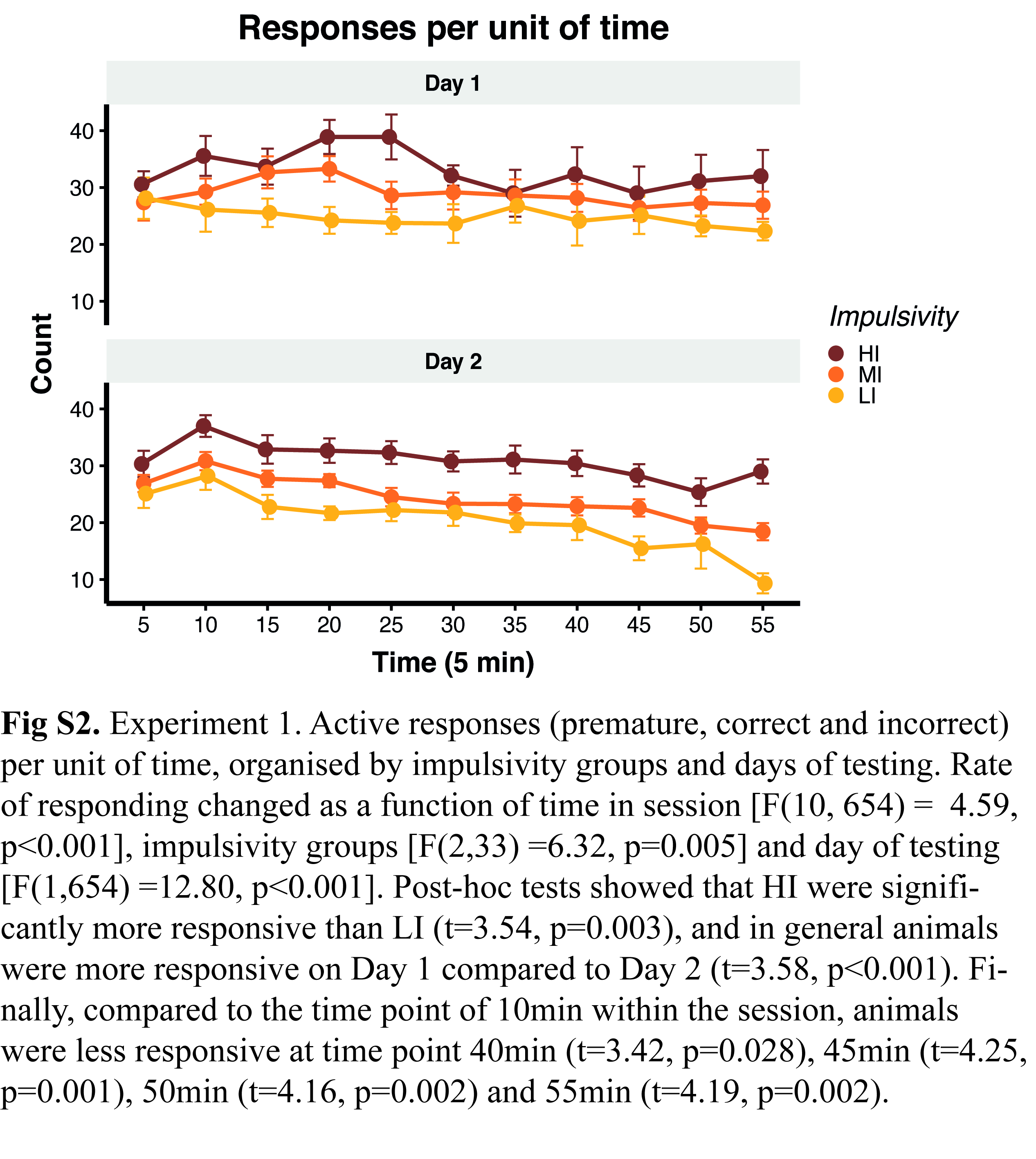

Supplement: Supplementary file 4 — High resolution image (TIF 29963 kb) [file 213_2021_5883_MOESM2_ESM.tif]

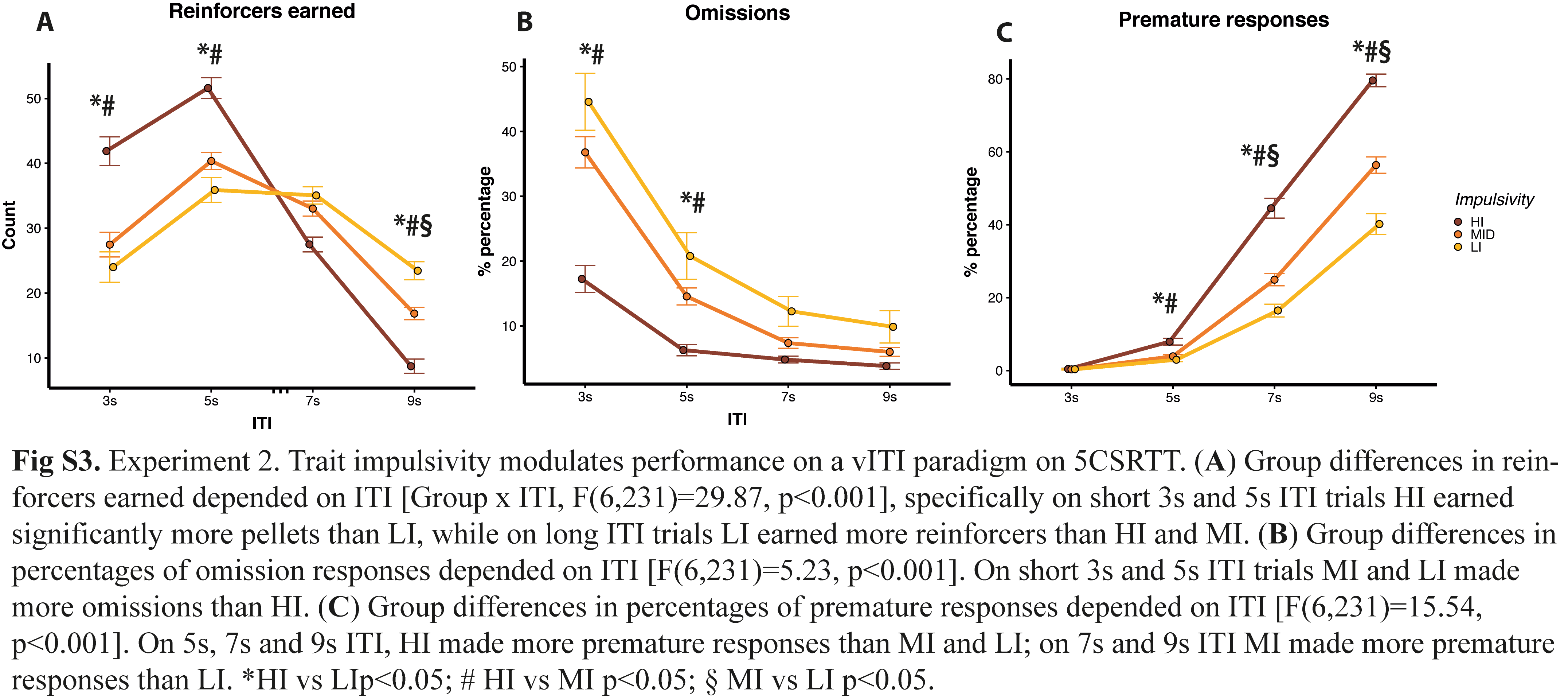

Supplement: Supplementary file 5 — (PNG 322 kb) [file 213_2021_5883_Fig3_ESM.png]

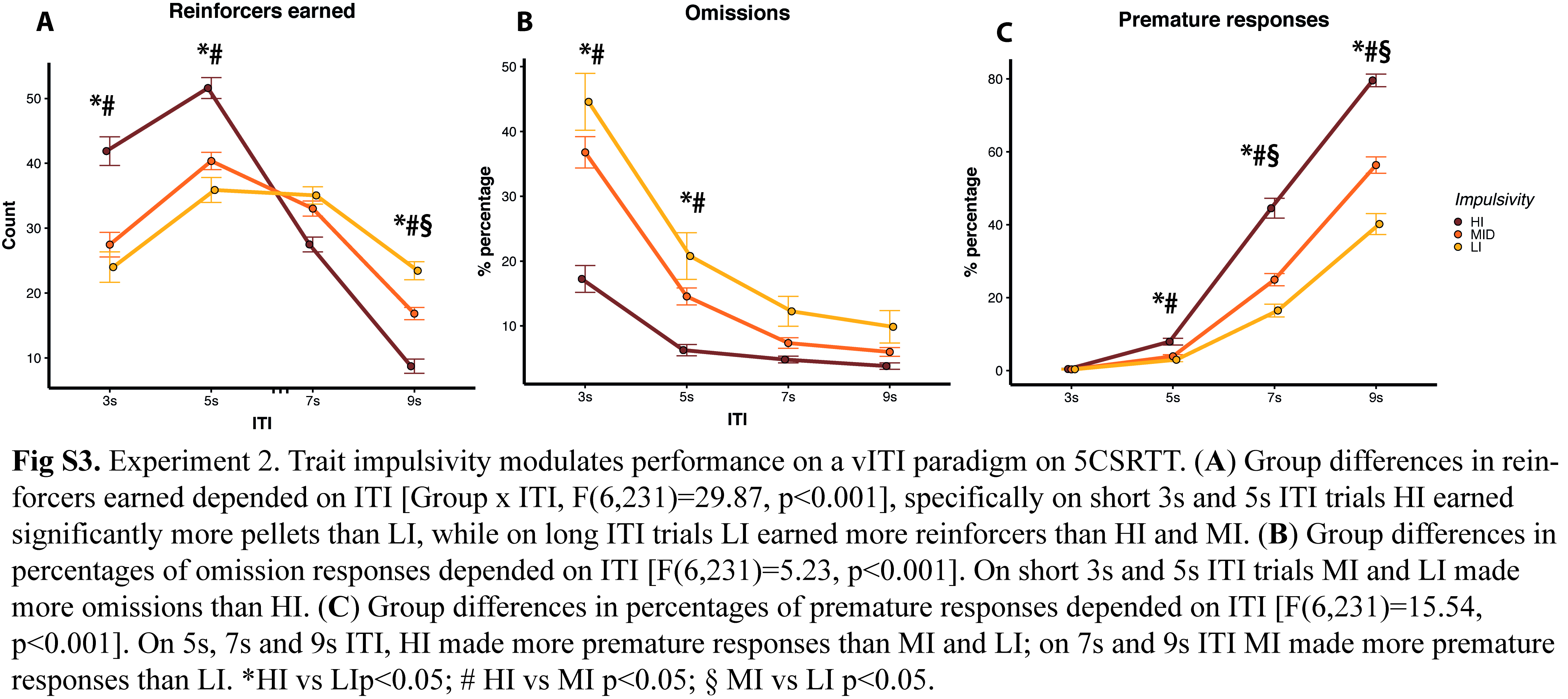

Supplement: Supplementary file 6 — High resolution image (TIF 34252 kb) [file 213_2021_5883_MOESM3_ESM.tif]

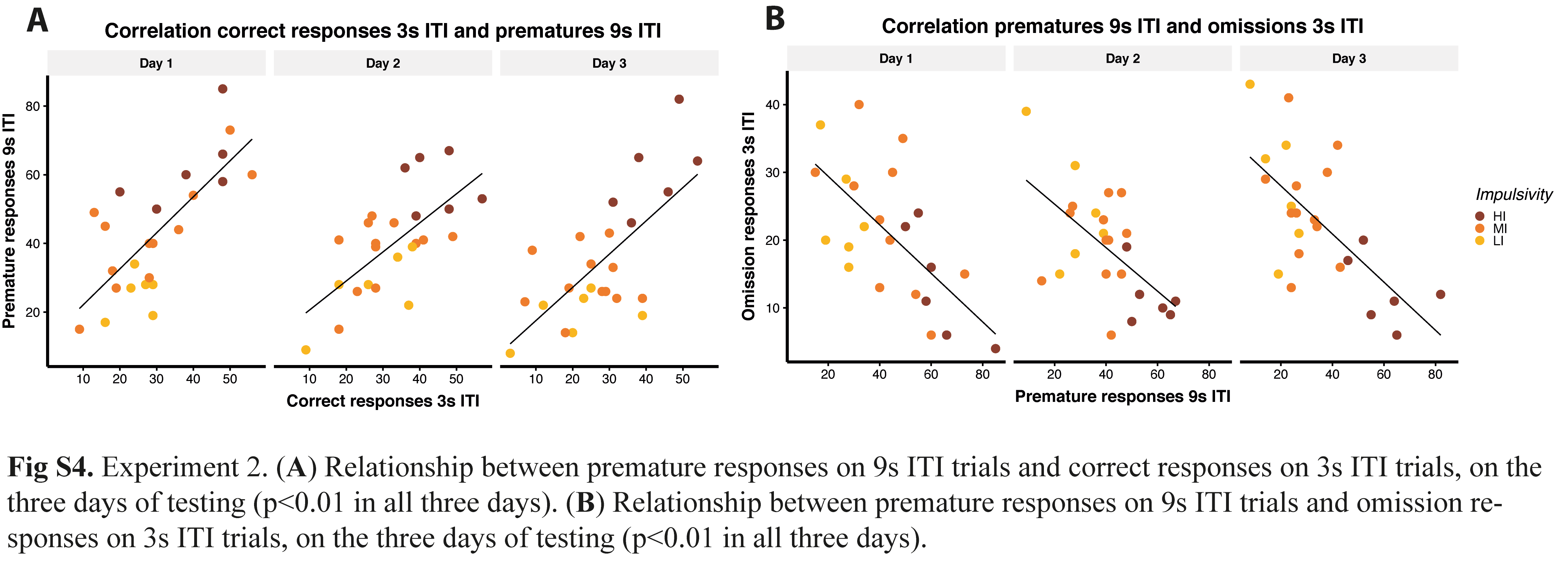

Supplement: Supplementary file 7 — (PNG 207 kb) [file 213_2021_5883_Fig4_ESM.png]

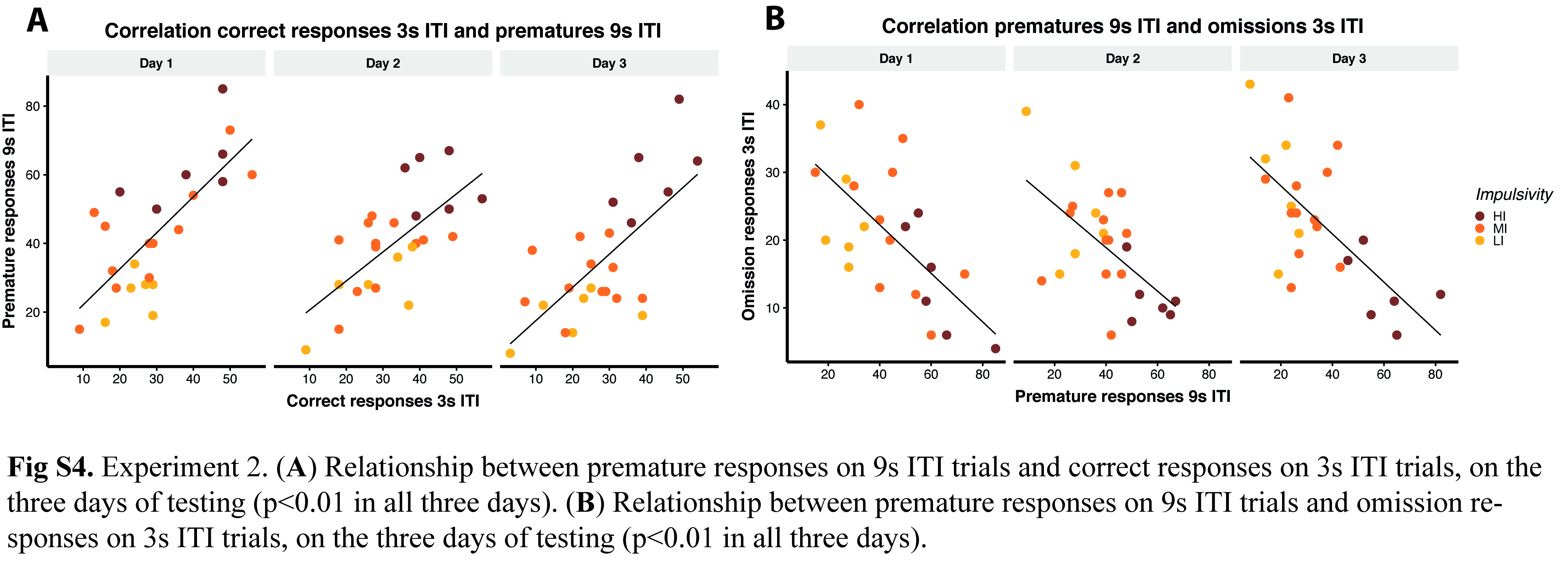

Supplement: Supplementary file 8 — High resolution image (TIF 29239 kb) [file 213_2021_5883_MOESM4_ESM.tif]

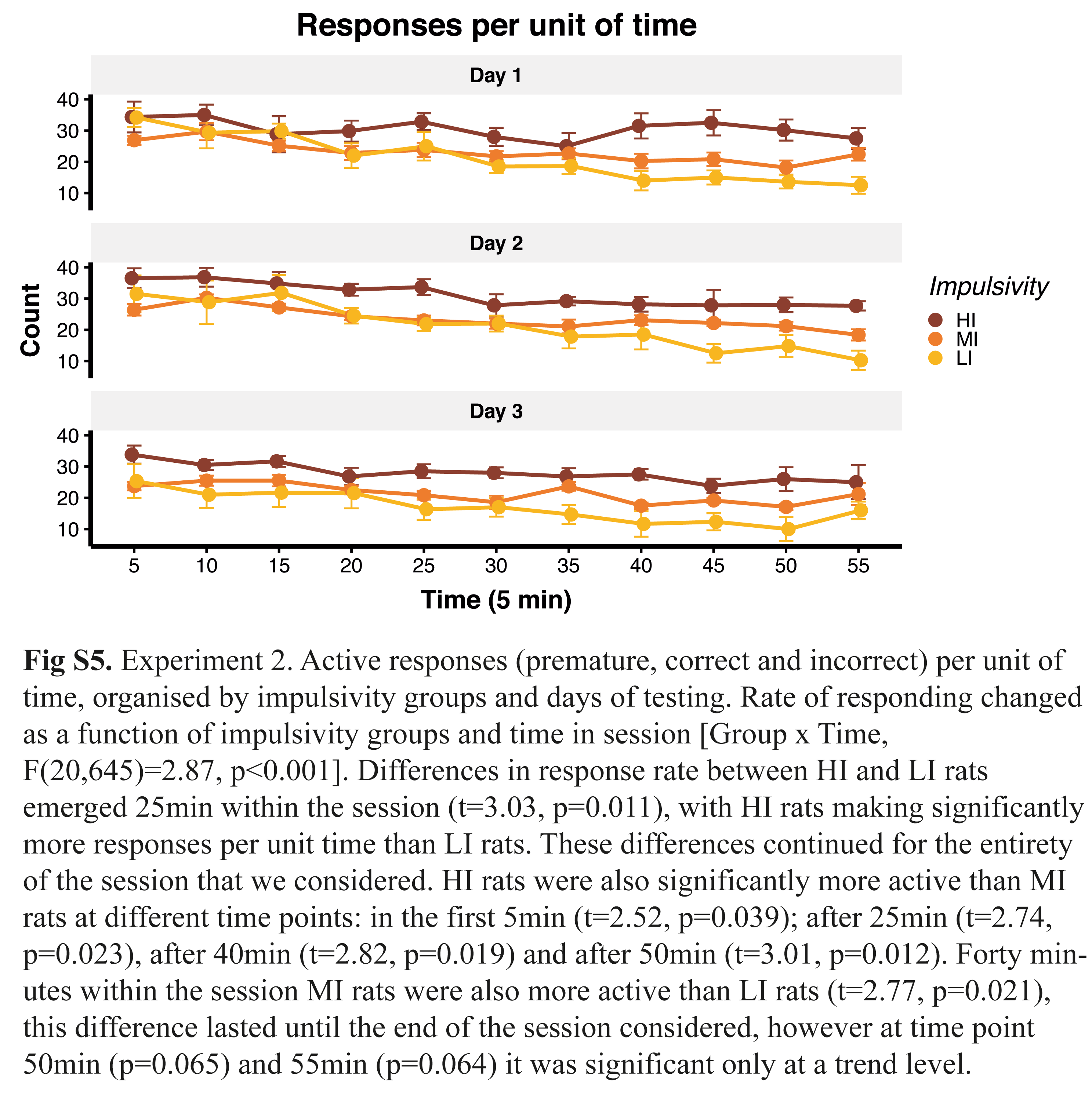

Supplement: Supplementary file 9 — (PNG 605 kb) [file 213_2021_5883_Fig5_ESM.png]

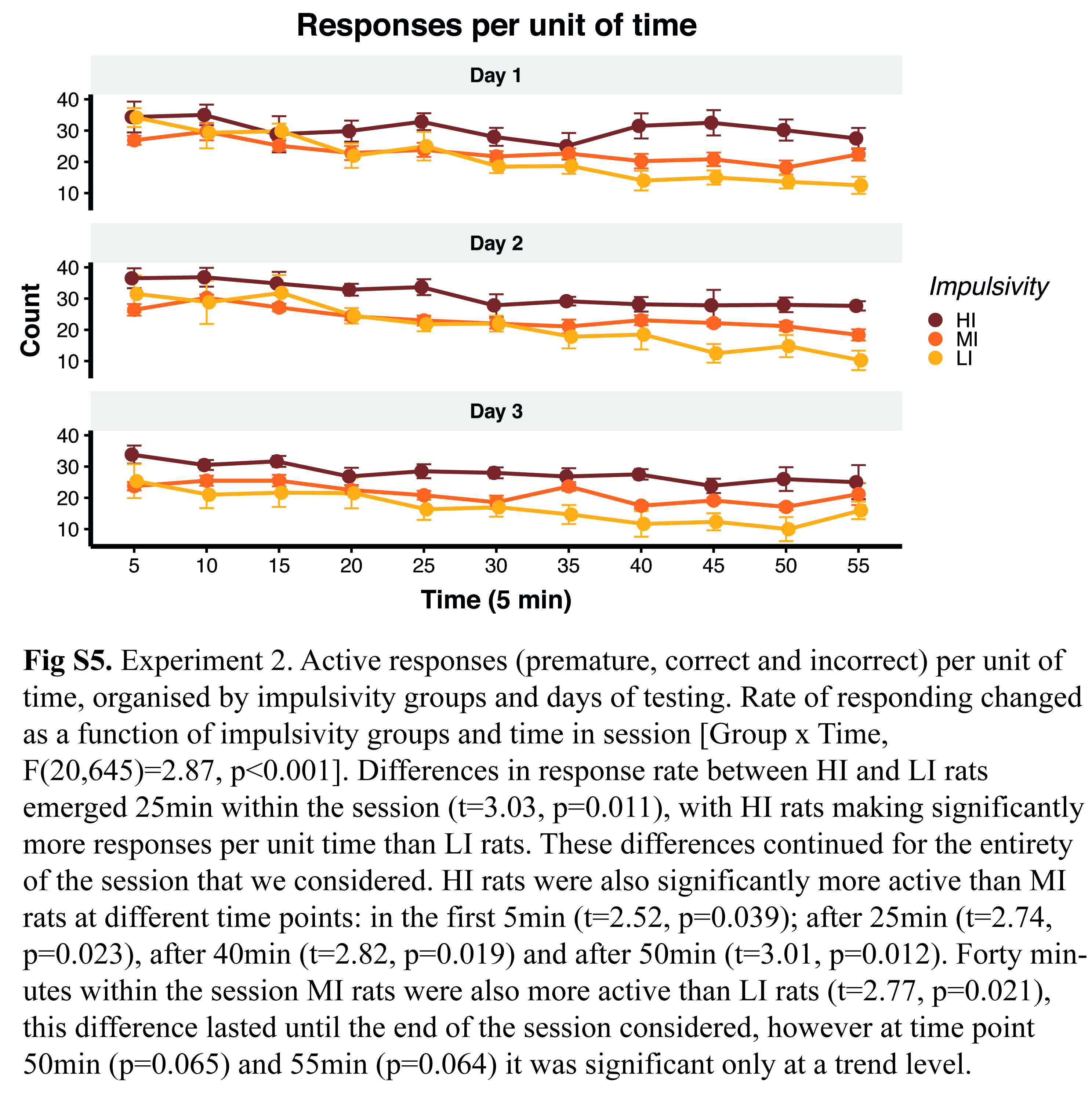

Supplement: Supplementary file 10 — High resolution image (TIF 37388 kb) [file 213_2021_5883_MOESM5_ESM.tif]

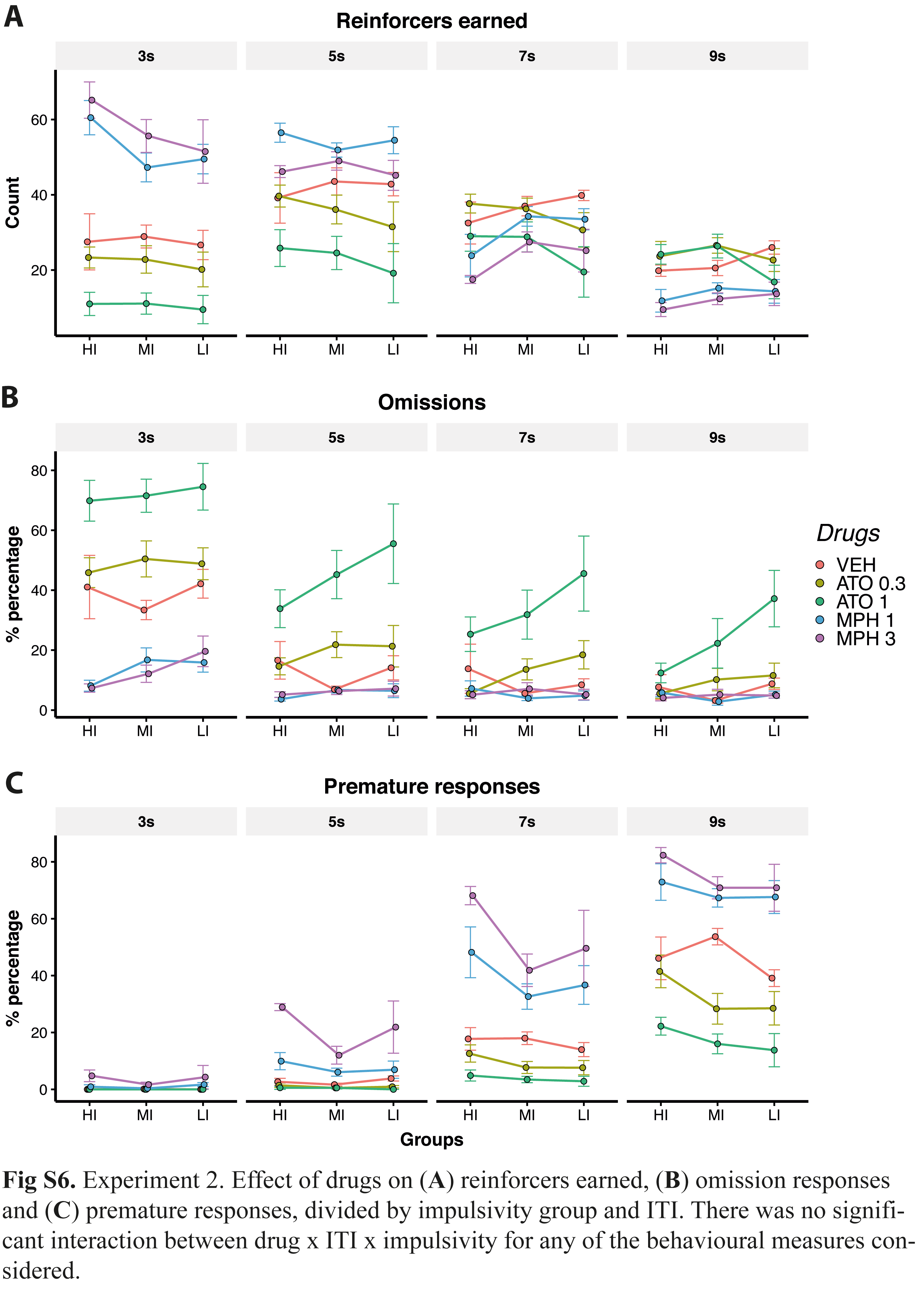

Supplement: Supplementary file 11 — (PNG 566 kb) [file 213_2021_5883_Fig6_ESM.png]

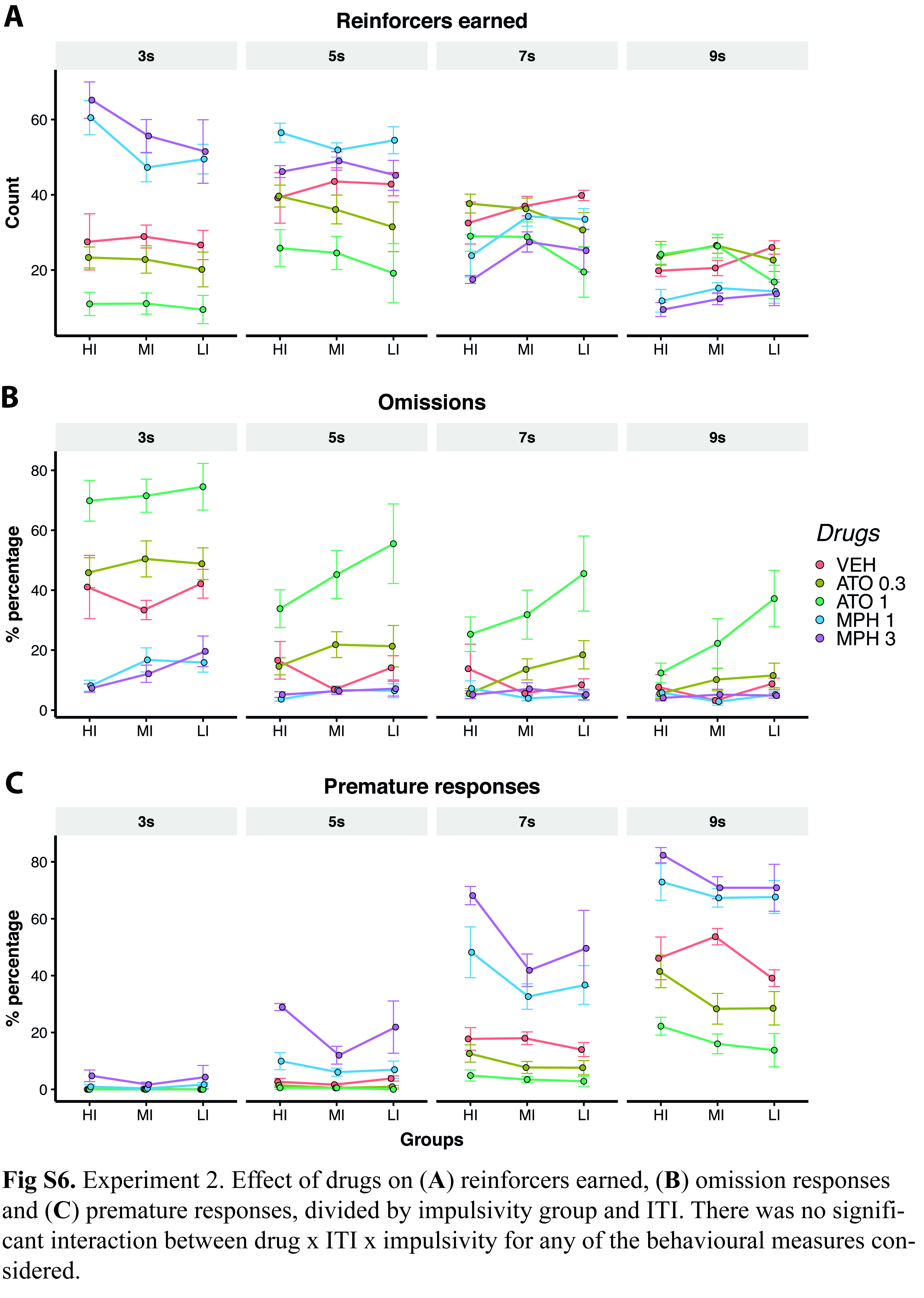

Supplement: Supplementary file 12 — High resolution image (TIF 49003 kb) [file 213_2021_5883_MOESM6_ESM.tif]

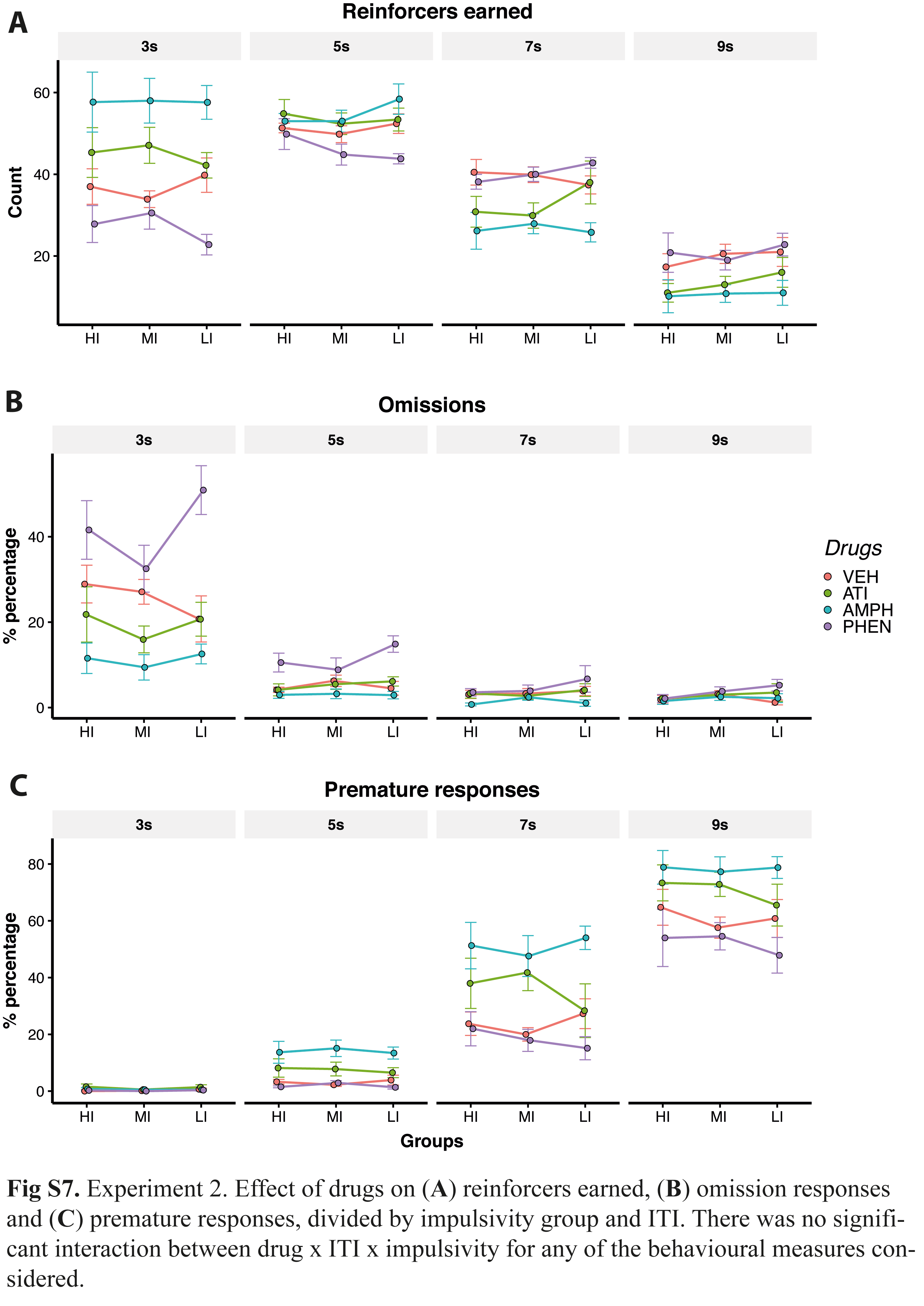

Supplement: Supplementary file 13 — (PNG 473 kb) [file 213_2021_5883_Fig7_ESM.png]

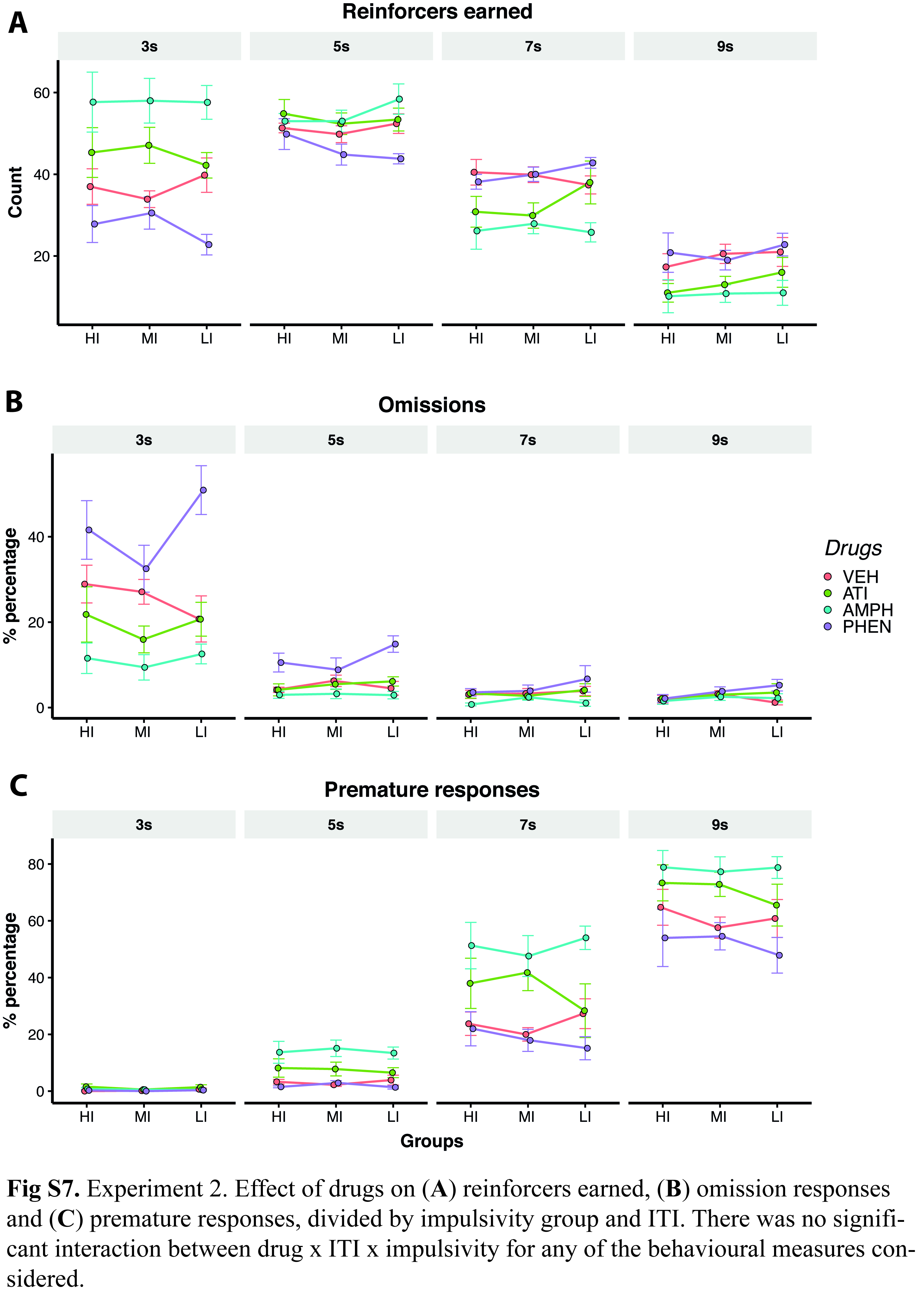

Supplement: Supplementary file 14 — High resolution image (TIF 49621 kb) [file 213_2021_5883_MOESM7_ESM.tif]
