# Supplementary material for: Adaptive aspects of impulsivity and interactions with effects of catecholaminergic agents in the 5-choice serial reaction time task: implications for ADHD
Source: Psychopharmacology (Berl). 2021 Jun 9;238(9):2601–15. doi: 10.1007/s00213-021-05883-y (PMC8373759; doi:10.1007/s00213-021-05883-y)
Supplement: Supplementary file 15 — (DOCX 31 kb) [file 213_2021_5883_MOESM8_ESM.docx]

SUPPLEMENTARY INFORMATION

**Adaptive aspects of impulsivity and interactions with effects of catecholaminergic agents in the 5-choice serial reaction time task: implications for ADHD**

Chiara Toschi^1^†, Mona El-Sayed Hervig^1,2^†, Parisa Moazen1,3, Maximillian Parker^1^, Jeffrey W Dalley^1,4^, Ulrik Gether^2^, Trevor W Robbins^1^

^1^Department of Psychology and Behavioural and Clinical Neuroscience Institute, University of Cambridge, Cambridge, UK

^2^Department of Neuroscience, University of Copenhagen, DK-2200 Copenhagen, Denmark

^3^Department of Physiology, Faculty of Medical Sciences, Tarbiat Modares University, Tehran, Iran

^4^Department of Psychiatry, University of Cambridge, Cambridge Biomedical Campus, Cambridge, UK

†These authors contributed equally to the work

**Corresponding author**: Chiara Toschi, [ct452@cam.ac.uk](mailto:ct452@cam.ac.uk), Behavioural and Clinical Neuroscience Institute Department of Psychology, University of Cambridge, Downing St., Cambridge CB2 3EB, Tel 01223-333550

**Training and baseline performance prior to the vITI challenge for Experiment 1 and Experiment 2**

There were no differences between groups with regards to number of sessions needed to reach baseline (prior to the vITI session), neither for Experiment 1 nor for Experiment 2 (p>0.05, for both cohorts). Analysis of the last baseline session prior to the vITI challenge revealed that, both in Experiment 1 and in Experiment 2, HI rats had elevated premature responses in this session compared to LI and MI rats. Specifically, for Experiment 1, impulsivity influenced the % of premature responses [F(2,33)=5.09 p=0.012], with HI rats making more premature responses than MI rats (p=0.010) and LI rats (0.07, trend level). Trait impulsivity also influenced % of premature responses in the baseline session prior to the vITI challenge of Experiment 2 [F(2,20)=4.19, p=0.031], with HI rats making more premature responses than LI rats (p=0.009). In this cohort of animals, impulsivity phenotype influenced performance accuracy during the baseline session [F(2,20)=3.75, p=0.041], with HI rats being slightly less accurate than LI rats (p=0.036).

**Assessment of impulsivity using the standard fixed 7s ITI paradigm. Experiment 2.**

Cohort 2 was assessed using a single fixed 7s ITI session to enable comparisons to be made with previous studies. A main effect of impulsivity phenotype [F(2,19)=12.02, p<0.001] revealed that HI rats categorized using the variable ITI procedure, exhibited significantly increased levels of premature responding compared with LI (p<0.001) and MI rats (p<0.001) during a fixed 7s ITI session.

|  | **Correct responses** | | **Incorrect responses** | |
| --- | --- | --- | --- | --- |
|  | **2s** | **3s** | **2s** | **3s** |
| **HI** | **1120±166.4*** | **800.2±60.1*** | 3227.1±197.2 | 2871±169.3 |
| **MI** | 1318.6±71.9 | 1009.8±64 | 3213.2±151.1 | 2596.7±131.2 |
| **LI** | **1616.3±221.6*** | **1169.1±119.2*** | 2994.5±170 | 2852.2±106.8 |

**Table S1.** Experiment 1, short vITI challenge. Latencies for correct and incorrect responses. *****HI vs LI p<0.05; **°** HI vs MID p<0.05.

|  | **Correct responses** | | | | **Incorrect responses** | | | | **Premature responses** | |
| --- | --- | --- | --- | --- | --- | --- | --- | --- | --- | --- |
|  | **3s** | **5s** | **7s** | **9s** | **3s** | **5s** | **7s** | **9s** | **7s** | **9s** |
| HI | 1145±67.9 | **685.3±24.2°*** | **567.4±16.8°*** | 622.1±40.7 | 2668.3±66.4 | 1690±97.9 | 1039.8±104 | 1369.6±202.3 | **5823.3±49°*** | **6786±74.3°*** |
| MI | 1367.2±77.9 | **930.9±42.7°** | **794.6±35.4°** | 770.3±33.4 | 3267.8±79.2 | 2114±84.4 | 1426.1±89.7 | 1262.5±100.6 | **5911.1±48.9°** | **7123.2±52.8°** |
| LI | 1109.9±63.8 | **909.5±41.8*** | **790.9±33*** | 704.5±31.7 | 3049.7±125.7 | 2281.2±136.6 | 1542.3±159.5 | 1335.1±178.8 | **6039.9±81.6*** | **7242.1±67.9*** |

**Table S2.** Experiment 2, vITI challenge. Latencies for correct, incorrect and premature responses. *****HI vs LI p<0.05; **°** HI vs MID p<0.05.

|  | **Correct responses** | | | | **Incorrect responses** | | | | **Premature responses** | |
| --- | --- | --- | --- | --- | --- | --- | --- | --- | --- | --- |
|  | **3s** | **5s** | **7s** | **9s** | **3s** | **5s** | **7s** | **9s** | **7s** | **9s** |
| **Veh** | 1159.5±53.5 | 858±58.2 | 669.6±30.3 | 689.5±43.3 | 3294.8±119.8 | 2167.7±149.5 | 1494.6±179.5 | 1005.4±126.1 | 5997.5±51.4 | 7341.8±55 |
| **ATO 0.3mg/kg** | 1175.5±75.1 | 893±46.6 | 715.2±26.5 | 665.5±26.8 | 3272.1±172.1 | 2313.2±153.2 | 1611.9±128.4 | 1098.6±81.4 | 6226.7±87.8 | 7472.8±93.6 |
| **ATO 1mg/kg** | **1451±134.5*** | **1046±**  **61.6*** | **812.4±**  **44.7*** | 756.2±33 | 3420.0±227.9 | 2718.8±207.2 | **2254.9±163.3*** | **1477.9±102.4*** | **6349.1±103.3*** | 7584.9±89 |

**Table S3.** Experiment 2, effects of ATO on a vITI challenge. Latencies for correct, incorrect and premature responses. ***** p<0.05 compared to vehicle.

|  | **Correct responses** | | | | **Incorrect responses** | | | |
| --- | --- | --- | --- | --- | --- | --- | --- | --- |
|  | **3s** | **5s** | **7s** | **9s** | **3s** | **5s** | **7s** | **9s** |
| **Veh** | 1159.5±53.5 | 858±58.2 | 669.6±30.3 | 689.5±43.3 | 3294.8±119.8 | 2167.7±149.5 | 1494.7±179.5 | 1005.4±126.1 |
| **Mph 1mg/kg** | **802±33.1*** | **638.6±21.1*** | 581.5±24 | 595±43.6 | 2633.4±151.1 | 1792.8±115.3 | 1146.2±125 | 1166.8±123.8 |
| **Mph 3mg/kg** | **763.6±29.8*** | **653.9±26.4*** | 675.5±43.9 | 678.7±62.3 | 2417.7±109 | **1433.9±118.9*** | 1548±210.3 | **1617.7±235.3*** |

**Table S4.** Experiment 2, effects of methylphenidate on a vITI challenge. Latencies for correct and incorrect. ***** p<0.05 compared to vehicle.

|  | **Veh** | | **ATO 0.3mg/kg** | | **ATO 1mg.kg** | | **MPH 1mg/kg** | | **MPH 3mg/kg** | |
| --- | --- | --- | --- | --- | --- | --- | --- | --- | --- | --- |
|  | **7s** | **9s** | **7s** | **9s** | **7s** | **9s** | **7s** | **9s** | **7s** | **9s** |
| **HI** | 5812.94±  93.19 | 7342.90±  157.16 | 6184.22±  168.09 | 7351.37±  217.73 | **6460.30±**  **23.77*** | 7667.88±  151.02 | 5589±  153.09 | **6486.39±**  **198.79*** | **5189.33±**  **82.84*** | **5544.52±**  **74.31*** |
| **MI** | 6041.73±  71.67 | 7369.17±  75.23 | 6402.32±  101.18 | 7409.92±  119.22 | **6186.54±**  **166.99*** | 7551.02±  144.81 | 5715.71±  147.41 | **7009.12±**  **138.06*** | **5648.50±**  **127.49*** | **6451.02±**  **203.75*** |
| **LI** | 6070.19±  92.96 | 7290.62±  69.19 | 5895.34±  164.21 | 7709.53±  180.33 | **4756.56±**  **1867.97*** | 7520.42±  166.57 | 5577.75±  237.71 | 6976.86±  223.81 | 5355.53±  330.74 | 6278.83±  524.27 |

**Table S5.** Experiment 2, effects of ATO and MPH on latency to make a premature response on a vITI challenge depended on the impulsivity group. ***** p<0.05 compared to vehicle.

|  | **Correct responses** | | | | **Incorrect responses** | | | | **Premature responses** | |
| --- | --- | --- | --- | --- | --- | --- | --- | --- | --- | --- |
|  | **3s** | **5s** | **7s** | **9s** | **3s** | **5s** | **7s** | **9s** | **7s** | **9s** |
| Veh | 981.8±46.6 | 714.8±29 | 564.3±17 | 548.9±22.3 | 3076.7±157 | 1974.3±163.6 | 1246.6±106.6 | 1045±127 | 6104.8±61.4 | 7305.2±72.4 |
| Amph 0.2mg/kg | **763.8±31.6*** | 633.3±28.1 | 549±24.8 | 641.1±42.6 | 2827.1±139.2 | 1410.1±129.8 | 1051±117.3 | 1038±133.2 | **5630±79.9*** | **6590.5±104.5*** |
| Ati 0.3mg/kg | **842.4±32.2*** | 670±39.6 | 568±22.6 | 553.8±35.9 | 3120±142.3 | 1537.4±162.9 | 1190.6±120.6 | 1215.4±146.2 | **5860.3±63.3*** | **6997.1±103.1*** |
| Phen 1mg/kg | 999.9±42.5 | 737.4±21.6 | 608.31±18.8 | **635.6±33.5*** | 3488.9±123.4 | 2060.4±141.7 | 1127.9±135.5 | 917.6±98.4 | 6114.2±75.1 | 7377.2±59.3 |

**Table S6.** Experiment 2, effects of AMPH, ATI and PHEN on a vITI challenge. Latencies for correct, incorrect and premature responses. ***** p<0.05 compared to vehicle.
